# Detection and Imaging of the Plant Pathogen Response by Near‐Infrared Fluorescent Polyphenol Sensors

**DOI:** 10.1002/anie.202108373

**Published:** 2021-11-22

**Authors:** Robert Nißler, Andrea T. Müller, Frederike Dohrman, Larissa Kurth, Han Li, Eric G. Cosio, Benjamin S. Flavel, Juan Pablo Giraldo, Axel Mithöfer, Sebastian Kruss

**Affiliations:** ^1^ Physical Chemistry II Bochum University Universitätsstrasse 150 44801 Bochum Germany; ^2^ Institute of Physical Chemistry Georg-August Universität Göttingen Tammannstrasse 6 37077 Göttingen Germany; ^3^ Research Group Plant Defense Physiology Max Planck Institute for Chemical Ecology Hans-Knöll-Strasse 8 07745 Jena Germany; ^4^ Institute of Nanotechnology Karlsruhe Institute of Technology (KIT) 76344 Eggenstein-Leopoldshafen Germany; ^5^ Institute for Nature Earth and Energy (INTE-PUCP) Pontifical Catholic University of Peru Av. Universitaria 1801, San Miguel 15088 Lima Peru; ^6^ Department of Botany and Plant Sciences University of California Riverside CA 92507 USA; ^7^ Fraunhofer Institute for Microelectronic Circuits and Systems Finkenstrasse 61 47057 Duisburg Germany

**Keywords:** biosensors, carbon nanotubes, imaging, near-infrared fluorescence, plant polyphenols

## Abstract

Plants use secondary metabolites such as polyphenols for chemical defense against pathogens and herbivores. Despite their importance in plant pathogen interactions and tolerance to diseases, it remains challenging to detect polyphenols in complex plant tissues. Here, we create molecular sensors for plant polyphenol imaging that are based on near‐infrared (NIR) fluorescent single‐wall carbon nanotubes (SWCNTs). We identified polyethylene glycol–phospholipids that render (6,5)‐SWCNTs sensitive (K_d_=90 nM) to plant polyphenols (tannins, flavonoids, …), which red‐shift (up to 20 nm) and quench their emission (ca. 1000 nm). These sensors report changes in total polyphenol level after herbivore or pathogen challenge in crop plant systems (Soybean Glycine max) and leaf tissue extracts (Tococa spp.). We furthermore demonstrate remote chemical imaging of pathogen‐induced polyphenol release from roots of soybean seedlings over the time course of 24 h. This approach allows in situ visualization and understanding of the chemical plant defense in real time and paves the way for plant phenotyping for optimized polyphenol secretion.

## Graphical abstract

Molecular sensors for plant polyphenol imaging based on near‐infrared (NIR) fluorescent single‐wall carbon nanotubes (SWCNTs) are presented. These sensors probe the polyphenol content in complex biological systems such as the plant rhizosphere. In summary, this approach enables real‐time spatiotemporal visualization of plant defense via polyphenols release.

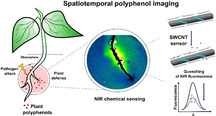

## Introduction

Smart agricultural solutions are required to optimize production practices and crop yields to enable a sustainable food supply for a rising global population. The rapid growth in human population will require a 60 % increase or more in food production by 2050 relative to 2005–2007 levels.^[1]^ In contrast, pathogen‐induced stresses significantly reduce crop health and yield.[^2^, ^3^] One solution is precision agriculture that aims for early detection of crop disease using vehicle remote imaging or sensing^[4]^ and crop phenotyping aims to breed plants with improved tolerance to pathogen stress. Tools for quantifying plants’ internal chemical signals associated with stress in real‐time are needed to boost these agriculture and phenotyping efforts.[^5^, ^6^]

Recent advances in chemistry and nanotechnology can contribute to improve crop production via novel sensor approaches allowing for remote analysis and optimization of plant traits.[^7^, ^8^] Semiconducting single‐wall carbon nanotubes (SWCNTs) are a powerful building block for these plant sensors because of their distinct photophysical properties.[^9^, ^10^, ^11^] Particularly, they fluoresce in the near‐infrared (NIR) region of the electromagnetic spectrum, which corresponds to the optical tissue transparency window due to decreased scattering and ultra‐low background.^[12]^ The discrete emission wavelength ranges from around 850 to 2400 nm and depends on their carbon lattice (band gap) structure, determined by the chirality (*n*,*m*).^[13]^ SWCNTs are not prone to photobleaching and can be used as optical labels and sensors, which are sensitive to their chemical environment.[^14^, ^15^, ^16^] SWCNT‐based sensors have been used as powerful imaging tools to analyze biological processes with high spatiotemporal resolution.[^17^, ^18^, ^19^] This technique was applied to detect genetic material,^[20]^ proteins,[^21^, ^22^] lipids,^[23]^ bacterial motives^[24]^ or small signaling molecules such as neurotransmitters,[^19^, ^25^] reactive oxygen species (ROS)[^26^, ^27^, ^28^] or nitric oxide (NO).^[29]^ More recently, their utilization as non‐genetically encoded sensors enabled the visualization of ROS patterns,[^27^, ^30^, ^31^] auxins^[32]^ or heavy metal uptake^[33]^ in plants.^[34]^ To tailor the SWCNT‐sensor properties, different chemical design strategies for surface functionalization have been developed. Most commonly, biopolymers such as single‐stranded (ss)DNA are adsorbed on the SWCNT surface, which mediates colloidal stability in aqueous solution and molecular recognition of the analyte.[^35^, ^36^, ^37^, ^38^] Other design and sensing concepts rely on non‐covalent functionalization with aptamers,[^17^, ^39^] phospholipids,^[40]^ peptides,[^41^, ^42^] proteins,^[43]^ or peptide–DNA hybrids.[^44^, ^45^] Recently, also covalent modification of fluorescent SWCNTs with (bio)molecules has been reported.[^46^, ^47^]

Detecting dynamic physiological processes in plants, such as defense responses to pathogen attack, could improve our understanding of plant pathogen interactions and help breed plants with increased biotic stress tolerance. Polyphenols, ubiquitous in the plant kingdom, are a prominent class of plant secondary metabolites involved in the constitutive and also inducible defense against pathogens and herbivores.[^48^, ^49^] They can be generally found in all plant tissues and organs and comprise a great variety of chemical structures with diverse ecological roles.^[50]^ One distinct aspect of polyphenol‐related plant defense is chemical secretion into the rhizosphere (root exudates), which modulates plant interactions within the soil ecosystem.^[51]^ These exudates/secretions are able to repel, inhibit, or even kill pathogenic microorganisms.^[51]^ Hence, increased production improves natural plant defense and is a goal of plant breeding.^[52]^

However, in situ detection and visualization of these biological processes remain a challenge because most analytical approaches cannot non‐invasively access in vivo systems.[^53^, ^54^, ^55^] Here, we created a NIR‐fluorescent sensor/probe for plant polyphenol detection and imaging. It responds to polyphenols in vitro and enables in vivo*/*in situ chemical imaging of polyphenols released from plant roots challenged with pathogen‐related stress.

## Results and Discussion

**Figure 1:**
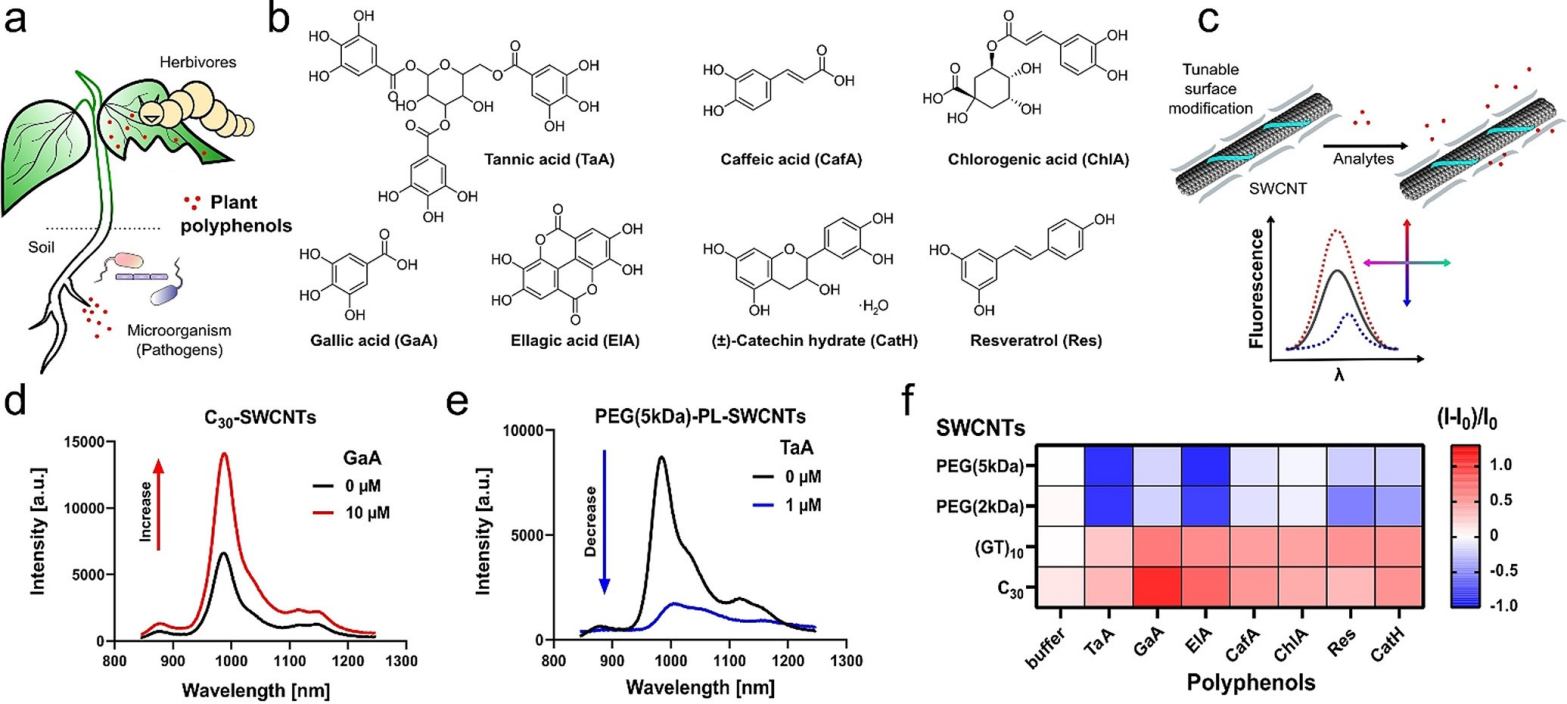
NIR fluorescent nanosensors for plant polyphenols. a) Plant polyphenols are released from leaves and roots in response to pathogens or herbivores and play an important role in chemical plant defense. b) Selected plant polyphenols investigated in this study. The compounds represent the subclasses of tannins, flavonoids and phenolic acids (see Supplementary Figure S1 for complete list). c) Non‐covalently modified SWCNTs with different kinds of biopolymers can change their fluorescence in response to polyphenols and serve as sensors by modulating emission intensity and energy (wavelength). d) NIR fluorescence spectra of single‐stranded (ss)DNA and e) PEG–phospholipid (PL)‐modified SWCNTs as examples for SWCNTs that change their fluorescence in response to polyphenols. f) Fluorescence change ((*I*−*I*_0_)/*I*_0_) of selected sensors in response to plant polyphenols (mean, *n*=3). Shades of blue indicate fluorescence decrease and shades of red fluorescence increase (polyphenol concentration=10 μM; TaA=1 μM).

Plant polyphenols are natural products with diverse chemical structures. Therefore, we tested how polyphenols (Figure 1 b) from different subgroups (e.g. tannins, flavonoids, phenolic acids) modulate the NIR fluorescence of SWCNT‐based molecular sensors. To assess the impact of surface chemistry (Figure 1) we used ssDNA with variable nucleotide composition (A,T,G,C) and polyethylene glycol (PEG)–phospholipid macromolecules for molecular assembly. Our rationale was that some of them have been known to interact with compounds that possess multiple hydroxy groups such as tannins.^[56]^ In general, the modified SWCNTs either increased or decreased their fluorescence in response to the target molecules, as shown for C_30_‐ and PEG(5 kDa)‐PL‐SWCNTs (Figure 1 c–e). For different SWCNT conjugates we observed changes in fluorescence (Figure 1 f) after addition of polyphenols in the physiologically relevant concentration range.[^50^, ^57^, ^58^] To exclude pH‐ or ionic strength‐related sensing effects,^[59]^ all experiments were performed in buffer. There is a general tendency of fluorescence increase for ssDNA‐SWCNTs and a decrease for PEG‐PL‐SWCNTs (see also Supplementary Figure S1). All tested compounds with two or more hydroxy residues on the phenol structure led to a significant fluorescence change, while salicylic acid did not alter the emission features of the tested SWCNTs. This finding was further supported by the lacking response of a trimethylated version of gallic acid (GaA) (Supplementary Figure S1).

**Figure 2:**
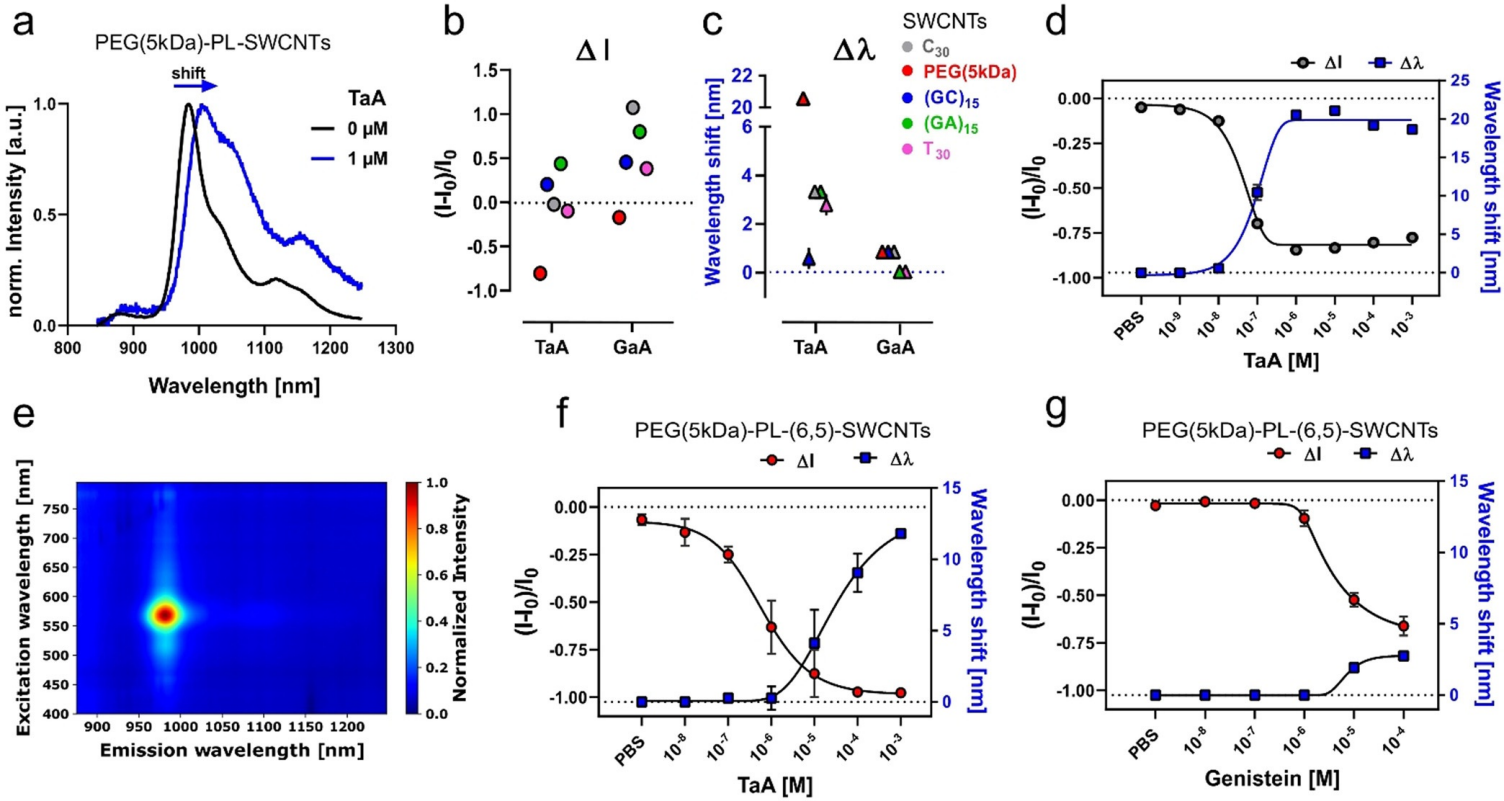
NIR detection of tannins in vitro. a) Normalized NIR fluorescence spectra of PEG‐PL‐SWCNTs without (black) and in presence (blue) of tannic acid (TaA). The emission wavelength shifts in addition to a change in fluorescence intensity. b) Comparison of intensity and in c) wavelength shifts of functionalized SWCNTs interacting with TaA and its subunits gallic acid (GaA) (10 μM; mean ± SD, *n*=3). Similar trends are visible for intensity changes, while emission wavelengths are not shifted in presence of GaA. It suggests that the 3D structure of TaA and less the gallic acid subunits are crucial. d) NIR fluorescence shifts of PEG‐PL‐SWCNTs in response to TaA. Intensity (black fit) decreases and wavelength shift (blue fit) increases in a concentration‐dependent manner (mean ± SD, *n*=3). e) 2D‐excitation emission photoluminescence spectra of chirality‐pure (6,5)‐SWCNTs. f) Monochiral sensor response of PEG‐PL‐(6,5)‐SWCNTs to TaA and g) to genistein addition. Intensity changes are indicated in red; wavelength shifts in blue (line=fit, mean ± SD, *n*=3).

Next to the evaluated intensity changes, also shifts in the emission wavelengths occurred. This phenomenon is most prominently observed for tannic acid (TaA) (Figure 2 a–c), resulting in ≈3 nm shifts for ssDNA‐ and ≈20 nm shifts for PEG‐PL‐SWCNTs. Only TaA caused a large emission wavelengths shift, whereas GaA changed ssDNA‐SWCNTs intensity more strongly (up to >100 %). These results suggest that the three‐dimensional structure of TaA affects sensing, an observation likely true also for other structurally large polyphenols. To better understand the interaction between tannins and the sensors, concentration‐dependent measurements were performed for different ssDNA‐SWCNTs. Interestingly, the fluorescence intensity increased in the nM regime, whereas it decreased for most sequences in the μM to mM range (Supplementary Figure S2). A uniform result was observed for PEG‐PL‐SWCNTs (Figure 2 a,d). Unlike for most ssDNA‐SWCNTs, the intensity decrease was clearly concentration dependent (*K_d_* =9.1×10^−8^ M) and saturated in the lower μM range (−80 % intensity change and ≈20 nm emission wavelength shift). GaA (1 mM) in contrast led to a much smaller sensor response of ≤−36 % and ≤3 nm shift. In addition to the change in emission of PEG‐PL‐SWCNTs also E_11_ absorption maximum was redshifted by ≈10 nm (Supplementary Figure S2). Overall, such interplay indicates a sensing mechanism based on a change in fluorescence quantum yield, without dominant aggregation effects. It furthermore suggests a strong interaction between sensor and analyte that goes beyond polyphenols acting as antioxidants[^60^, ^61^] and might include changes in solvation that affects exciton diffusion. Hence, PEG‐PL‐SWCNTs showed the most promising response to plant polyphenols.

Next, we evaluated if sensing with monochiral SWCNTs of a well‐defined emission wavelength (color) is possible. Non‐overlapping emission spectra are required for multiplexed sensing and hyperspectral imaging approaches. To obtain monochiral (6,5)‐SWCNTs, aqueous two‐phase separation (ATPE) was performed, followed by surface exchange to PEG–PL (Figure 2 e, Supplementary Figure S3). Monochiral sensors responded in a similar fashion (*K_d_* =4.3×10^−6^ M) (Figure 2 f). Similar sensor responses were observed for the isoflavonoid called genistein (Figure 2 g). It has been described that mainly surface modification imparts sensitivity and selectivity and not chirality.^[62]^ However, experiments with multi‐chirality (HiPco) PEG‐PL‐SWCNTs showed distinct differences pointing to a polyphenol profile and chirality‐dependent response (Supplementary Figure S4).

**Figure 3:**
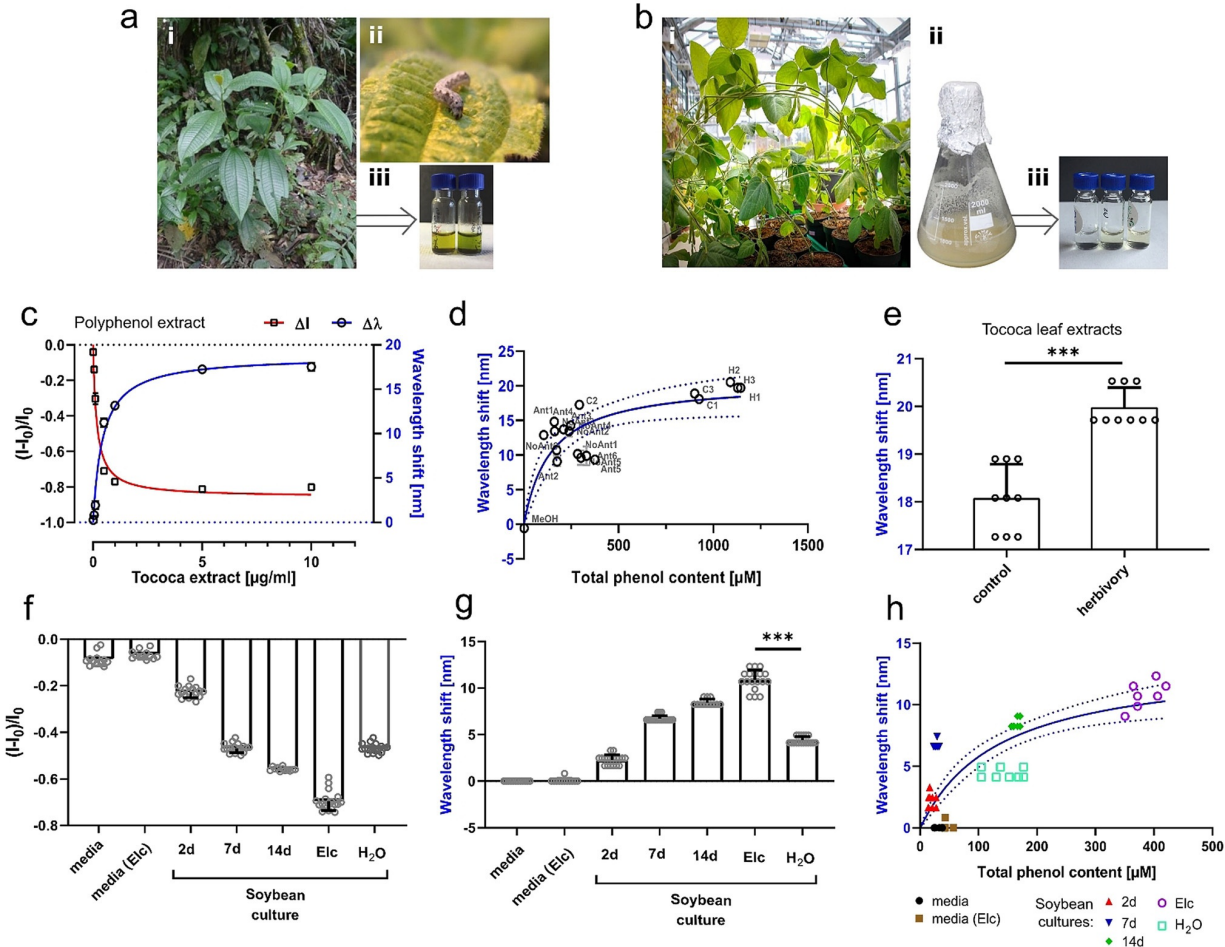
Polyphenol detection in plant extracts. a) *Tococa* spp. plants. i—wild plants found in the Peruvian rain forest. ii—the herbivore *Spodoptera littoralis* on a *Tococa* leaf. iii—crude MeOH leaf extracts used for sensor testing. b) Soybean (*Glycine max*) plants. i—an adult *G. max* plant grown in the greenhouse. ii—*G. max* suspension cell culture. iii—cell culture supernatant used for nanosensor testing. c) Nanosensor (PEG‐PL‐SWCNTs) response against purified polyphenol extract from *Tococa* spp., containing all extractable leaf polyphenols with a predominantly high ellagitannin content. The NIR fluorescence decreases and simultaneously the emission wavelength shifts (mean ± SD, *n*=3, colored line=hyperbolic fit). d) Correlation of emission wavelength shift and the total phenol content from multiple *Tococa* leaf MeOH extracts (measured using the Folin–Ciocalteu reagent, which is an established colometric assay). The dynamic range of the sensor is in the μM range (K_d_=142 μM, expressed as gallic acid equivalents) (mean ± SD, *N*=1, *n*=3, tested as 2 μL non‐diluted MeOH extracts, blue line=hyperbolic fit; C=control, H=herbivory, Ant=plants with ant symbionts). e) *Tococa* leaf extracts from plants challenged with herbivores (*S. littoralis*) give a significantly different nanosensor response compared to non‐treated plants (mean ± SD, *N*=3, *n*=3, unpaired t‐test). f) Soybean (*Glycine max*) cell culture samples decrease fluorescence and shift emission wavelengths (g) of nanosensors, which allows detection of age‐ and pathogen‐induced (Elc, elicitor) changes in polyphenol levels (mean ± SD, *N*=6, *n*=3,***P<0.001; one‐way ANOVA). h) Correlation of emission wavelength shift with total phenol content (quantified by Folin–Ciocalteu reagent) shows a hyperbolic trend with a *K*_d_ of 140 μM (*N*=6, *n*=2, blue line=hyperbolic fit).

To test these sensors in more complex environments we used plant tissue extract and culture medium. For this purpose, methanol extracts from *Tococa* spp. leaf tissue (Figure 3 a) and liquid media from soybean (*Glycine max*) suspension cell cultures (Figure 3 b) were tested. Neotropical *Tococa* spp. is known for its high polyphenol content (e.g. ellagitannins, Supplementary Figure S5) and serves as a model system for polyphenol releasing plants. The sensors showed a strong fluorescence decrease (Figure 3 c) along with a large emission shift in a low μg mL^−1^ range. This response correlates with the response of pure polyphenols (Figure 1 and Figure 2). The total phenol content was additionally quantified with an established colorimetric assay (Folin–Ciocalteu reagent^[63]^). The sensor responses (*K_d_* =1.5 μM for purified polyphenols, Supplementary Figure S5) correlated with total phenol content (expressed as gallic acid equivalents). When correlating all tested *Tococa* leaf methanol extracts, an overall curve fitting with *K_d_* ≈140 μM was obtained (Figure 3 d). These results indicate that the sensors are able to probe the species‐specific phenol content as relative increases even within a complex background (Supplementary Figure S6). Additionally, extracts of plants stressed by insect herbivory caused a significant difference in sensor response (Figure 3 e), which correlated with an increased total phenolic content (Figure 3 d). The results are in agreement with classical HPLC–MS polyphenol detection (Supplementary Figure S7) and demonstrate that these NIR fluorescent sensors identify polyphenols even with a chlorophyll or sugar background (methanol extraction). Plant extracts from the field (Peruvian rainforest in the Tambopata National Reserve) showed a similar response as HPLC–MS‐based detection (Supplementary Figure S7). Therefore, these nanosensors are a valuable tool for rapid and high‐throughput screening, requiring very small volume (few μL) of plant extracts. These hallmarks are desired for testing of plant analytes that are difficult to extract in large volumes, for example, phloem sap, or with low concentration of analytes, for example, xylem sap. The second plant system were soybean‐based (*Glycine max*) suspension cells (Figure 3 b, Supplementary Figure S8). They are known to release polyphenols, in particular pterocarpans, into the medium during aging or due to pathogen stress.[^64^, ^65^] We directly added the cell‐free supernatant of the culture to the nanosensors, without further purification. Mature cells showed a stronger sensor response, which means that they produced more polyphenols (Figure 3 f,g, Supplementary Figure S9).

These soybean cultures were also stimulated with a pathogen‐derived elicitor, a branched β‐glucan cell wall component of the Oomycete fungus *Phytophthora sojae*, which induces a defense‐related response that triggers secretion of polyphenols.[^66^, ^67^, ^68^] This elicitor (Elc) caused a significant sensor response (intensity changes and emission wavelength shift, Figure 3 f,g). Both the control (H_2_O) and the stimulated cultures were 7 days old, hence containing next to the elicitor‐induced polyphenols, pterocarpan derivates, also aging‐related ones like genistein. HPLC–MS analysis further confirmed the increase in polyphenols after elicitor stimulus (see Supplementary Figure S8 and S10). Furthermore, soybean defensive polyphenols genistein and trihydroxypterocarpan (THP) modulate the NIR fluorescence in a concentration‐dependent manner (Supplementary Figure S11). Together these results show that our sensor can report polyphenol release from plants or cells in vitro.

The sensor response to total phenol content of soybean cells (Figure 3 h) is hyperbolic (*K_d_* ≈140 μM) and is not biased by cell medium or elicitor (Figure 3 g,h). Even though there are differences in sensitivity toward different polyphenols, the presented PEG‐PL‐SWCNT is therefore a total polyphenol content sensor. A major advantage of a fluorescent sensor/probe is that it can be used for imaging and provide additional spatiotemporal information compared to standard analytical methods (e.g. HPLC/GC–MS, colorimetric assays, biosensors).[^53^, ^54^, ^55^, ^63^]

**Figure 4:**
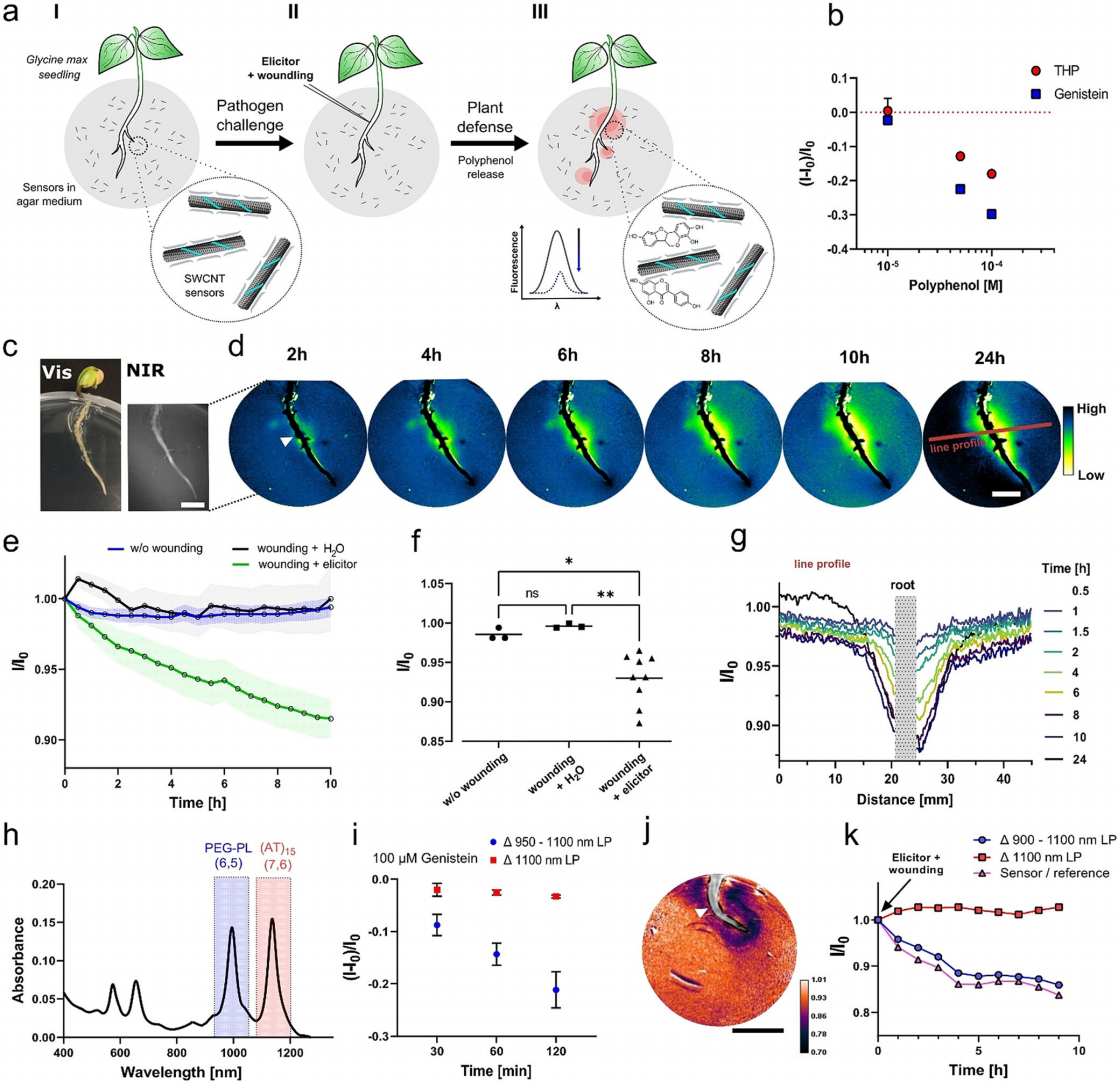
Real‐time imaging of pathogen‐induced polyphenol release from roots. a) Chemical imaging concept with SWCNT‐based fluorescent sensors incorporated in culture medium agar. Soybean seedling (*G. max*) grow through the agar. The plant is challenged with a pathogen elicitor and the response (polyphenol secretion) is monitored by NIR fluorescent stand‐off imaging (>20 cm). b) Genistein and trihydroxypterocarpan (THP) as prominent components of the soybean (*G. max*) polyphenol profile quench the fluorescence of PEG‐PL‐SWCNTs in agar (mean ± SD, *n*=3). c) Visible and NIR image of the soybean seedling (scale bar=1 cm). d) The NIR fluorescence of the sensors (*I*/*I*_0_) in the plant environment (rhizosphere) decreases over time close to the challenged root position (root tissue is overlayed with black; white triangle=position for elicitor induction; red line=line profile position, scale bar=1 cm). e) Sensor image (500‐pixel, ≈170 mm^2^) reports polyphenol release to a fungal elicitor. In contrast, the sensor does not respond in the absence of stimulus (without wounding) or to wounding + H_2_O (mean, error bars=SD, *n*=1). f) Sensor intensity changes 10 h after stimulus. Mean pixel intensities of 500‐pixel areas close to the challenged root position (mean, control and H_2_O *N*=3; elicitor *N*=9, *P<0.033; **P<0.002; ns=not significant; one‐way ANOVA). g) Spatiotemporal profile of the plant defense via polyphenol release (line profile for 5‐pixel width section shown in Figure 4 d). h) Absorbance spectra of monochiral (6,5)‐PEG–PL as polyphenol sensor and (AT)_15_‐(7,6)‐SWCNTs (reference) in agar. i) NIR stand‐off imaging of monochiral sensors and their response. The fluorescence of PEG‐PL‐(6,5)‐SWCNTs (950 nm long pass (LP) filter image − 1100 nm LP filter image) decreases in response to genistein (100 μM). In contrast, (AT)_15_–17,6‐SWCNTs (1100 LP filter) are not strongly affected and serve as a reference. j) Ratiometric image of challenged soybean seedling (*t*=9 h post induction, ratio Δ*H* (900 LP − 1100 LP)/(1100 LP), white triangle=position for elicitor induction, scale bar=1 cm). k) Ratiometric imaging of polyphenol release over time (sensor=PEG‐PL‐(6,5)‐SWCNTs: 900–1100 nm; reference=(AT)_15_‐(7,6)‐SWCNTs: >1100 nm) (mean, *N*=1) measured as mean pixel intensity (500‐pixel, ≈170 mm^2^) over time.

To image plant polyphenol secretion over time (Figure 4 a) we embedded the sensors in agar and let soybean seedlings grow on top. First, we had to optimize the conditions for embedding PEG‐PL‐SWCNTs into agar, as agar and salt concentration seemed to play an important role for photoluminescence and sensing (Supplementary Figure S12). The representative polyphenols genistein and THP were used to evaluate the sensing performance (Figure 4 b) and showed up to 30 % fluorescence decrease (100 μM) within 30 min. On the other hand, potential interfering substances from the root, such as sugars or H_2_O_2_, did not alter the fluorescence emission (Supplementary Figure S13). The plant defense by polyphenols was then imaged remotely in real‐time by a NIR stand‐off imaging^[24]^ system (Supplementary Figure S13). For this purpose, soybean seedlings were plated onto the optimized sensor agar (Figure 4 c) and the embryonic root was imaged for 24 h with elicitor stimulus or its respective control (Supplementary Video S1 and S2). The NIR (Figure 4 d) signal decreased close to the wound, indicating polyphenol secretions close to the elicitor‐induced root area, as hypothesized before.^[64]^ These results confirm studies with pathogen (*P. sojae)*‐infected soybean seedlings,[^69^, ^70^] performed by laborious and tissue‐destructive methods involving antibodies in combination with cryotome‐prepared root tissue sections. Polyphenol secretion and diffusion increase in the first 4–8 h and remain stable over the 24‐hour experimental timeframe (see also Supplementary Figure S14). When wounding the embryotic root and applying H_2_O instead of the elicitor, no enhanced nanosensor response was detected (Figure 4 e), which confirms that both mechanical wounding and a chemical elicitor are necessary similar to a pathogen attack.^[69]^ The difference in polyphenol secretion between individual plants (Figure 4 f) could be used to identify plant cultivars with improved pathogen response. The largest sensor fluorescence modulation occurred in close proximity to the embryotic root (see Figure 4 g), indicating higher changes of polyphenol content in this region of the rhizosphere. To further improve imaging we also implemented ratiometric sensing in which one sensor reports the analyte of interest at one wavelength and another sensor serves as reference at a different wavelength that is not affected by the analyte.^[30]^ For such an approach, (n,m) chirality‐pure or even enantiomer‐pure SWCNTs are necessary.^[62]^ We prepared chirality‐pure (6,5)‐SWCNTs with PEG–PL to act as polyphenol‐responsive sensor, while monochiral (7,6)‐SWCNTs coated with (AT)_15_‐ssDNA served as reference that does not react to polyphenols (Figure 4 h, Supplementary Figure S1, S15). Inside agar, they allowed ratiometric imaging using appropriate emission filters (PEG‐PL‐(6,5)‐SWCNTs: 900–1100 nm and (AT)_15_‐(7,6)‐SWCNTs: >1100 nm). This approach enabled ratiometric detection of the important soybean polyphenol genistein (Figure 4 i) and also ratiometric imaging of the elicitor‐induced secretion of root polyphenols (Figure 4 j,k, Supplementary Video S3). The concept could be expanded to multiplexing to study the co‐secretion of multiple plant‐defense molecules (exudates) and improve our understanding of spatiotemporal chemical processes in the complex rhizosphere.^[71]^ Additionally, this ratiometric approach is less prone to variations in the position of the light source and camera and therefore guarantees a more robust imaging concept with better signal/noise ratio.

## Conclusion

We have synthesized molecular sensors based on SWCNTs for NIR imaging of polyphenols. They allow to observe the response of plants to pathogens via release of polyphenols with high spatiotemporal resolution in the beneficial NIR tissue transparency window. The sensors probe the polyphenol content in complex biological systems such as the plant rhizosphere. This tool can be used to better understand plant chemical defense mechanisms as well as plant chemical communication and accelerate phenotyping and identification of crop plants that are more tolerant to pathogens.[^72^, ^73^, ^74^] We showcased the potential for a main crop plant species (soybean) and in plant extracts or tissue culture media without further purification. Sensor responses showed a strong correlation with classical analytical methods like colorimetric assays or HPLC–MS quantification but had the major advantage of in situ detection without further sample taking or handling. Additionally, the spatiotemporal resolution provided novel insights into the time scale and spatial dimensions of polyphenol secretion. Such rapid optical detection could be used for high‐throughput screening tools that require minimal plant sample volumes down to the μL scale or to remotely visualize pathogen‐induced plant defense and stress. In summary, this technique paves the way for precision agriculture and demonstrates the versatility of tailored nanoscale sensors for chemical imaging.

## Conflict of interest

The authors declare no conflict of interest.

## Supporting information

As a service to our authors and readers, this journal provides supporting information supplied by the authors. Such materials are peer reviewed and may be re‐organized for online delivery, but are not copy‐edited or typeset. Technical support issues arising from supporting information (other than missing files) should be addressed to the authors.

Supporting Information

Supporting Information

Supporting Information

Supporting Information
