# Supplementary material for: Detection and Imaging of the Plant Pathogen Response by Near‐Infrared Fluorescent Polyphenol Sensors
Source: Angew Chem Int Ed Engl. 2021 Nov 22;61(2):e202108373. doi: 10.1002/anie.202108373 (PMC9298901; doi:10.1002/anie.202108373)
Supplement: Supplementary file 1 — Supporting Information [file ANIE-61-0-s001.pdf]

Angewandte Chemie
Eine Zeitschrift der Gesellschaft Deutscher Chemiker

Supporting Information

Detection and Imaging of the Plant Pathogen Response by Near-Infrared Fluorescent Polyphenol Sensors

*Robert Nißler, Andrea T. Müller, Frederike Dohrman, Larissa Kurth, Han Li, Eric G. Cosio, Benjamin S. Flavel, Juan Pablo Giraldo, Axel Mithöfer, and Sebastian Kruss**

anie_202108373_sm_miscellaneous_information.pdf
anie_202108373_sm_SV1.avi
anie_202108373_sm_SV2.avi
anie_202108373_sm_SV3.avi

Table of Contents

Experimental Procedures **2**
SWCNT surface modification 2
NIR spectroscopy 2
SWCNT separation 2
NIR stand-off imaging 2
Plant material and classical polyphenol detection 2
Incorporation of *G. max* seedlings into NIR fluorescent agar 4
Polyphenol visualization 4
Image analysis 4
Results and Discussion **5**
Figure S1: Sensor screening for plant polyphenol detection 5
Figure S2: Sensing of tannic acid (TaA) 6
Figure S3: SWCNT purification to obtain monochiral PEG-PL-(6,5)-SWCNT sensors 7
Figure S4: Polyphenol interaction with HiPco-SWCNTs 8
Figure S5: Total phenol content quantification and analysis of *Tococa spp.* polyphenol extract 9
Figure S6: Total phenol content quantification and analysis of *Tococa spp.* polyphenol extract 9
Figure S7: Analysis of crude *Tococa spp.* leaf extracts 10
Figure S8: Characterization of polyphenols from soybean cell cultures 12
Figure S9: Sensor response to soybean cell cultures 13
Figure S10: Untargeted metabolomics of treated and untreated soybean cell cultures 14
Figure S11: Sensor response to prominent soybean polyphenols 15
Figure S12: Tuning the nanosensor incorporation into functional agar medium 16
Figure S13: Imaging of sensor response during agar diffusion 17
Figure S14: Elicitor induced polyphenol release from soybean seedlings 18
Figure S15: Separated SWCNT fractions for hyperspectral sensing 20
References **20**
Author Contributions **20**

Experimental Procedures

All materials, if not otherwise stated, were purchased from Sigma Aldrich.

SWCNT surface modification

(6,5)-chirality enriched CoMoCat SWCNTs (Sigma-Aldrich, product no. 773735) were modified with varying single-stranded (ss)DNA sequences such as $(GT)_{10}$, $(CT)_{15}$, $(GA)_{15}$, $(GC)_{15}$, $(AT)_{15}$, $(A)_{30}$, $(T)_{30}$ and $(C)_{30}$ (oligonucleotide sequences purchased by Sigma Aldrich) following a previously described protocol.[1] In short, 100 µL ssDNA (2 mg/mL in H_2O) were mixed with 100 µL 2xPBS and 100 µL SWCNTs (2 mg/mL in PBS), tip-sonicated for 15 min @ 30% amplitude (36 W output power, Fisher Scientific model 120 Sonic Dismembrator) and centrifuged 2x 30 min @ 16100x g.
Phospholipid-PEG-SWCNTs were assembled by performing dialysis of sodium cholate suspended SWCNTs.[2] Here, 2 mg SWCNTs (CoMoCat (Sigma-Aldrich, product no. 773735) or HiPco (NanoIntegris HiPco Raw SWCNTs)) were tip-sonicated in 750 µL sodium cholate (10 mg/mL in PBS). After centrifugation (2x 30 min @ 16100x g), 200 µL supernatant was mixed with 800 µL sodium cholate (12 mg/mL) containing 2 mg 18:0 PEG5000 PE (1,2-distearoyl-sn-glycero-3-phosphoethanolamine-N-[methoxy(polyethylene glycol)-5000]) or 2 mg DSPE-PEG(2000) Amine (1,2-distearoyl-sn-glycero-3-phosphoethanolamine-N-[amino(polyethylene glycol)-2000]), Avanti Lipids). The mixture was transferred to a 1 kDa dialysis bag (Spectra/Por®, Spectrum Laboratories Inc.) and dialyzed for several days (>5 d) against 1xPBS. The colloidally stable PEG-PL-SWCNTs sensors were obtained after centrifugation for 30 min @ 16100x *g.*

NIR spectroscopy

Absorption spectra were acquired with a JASCO V-670 device from 400 to 1350 nm in 0.2 nm steps in a 10 mm path glass-cuvette. 1D-NIR fluorescence spectra were measured with a Shamrock 193i spectrometer (Andor Technology Ltd., Belfast, Northern Ireland) connected to a IX73 Microscope (Olympus, Tokyo, Japan). Excitation was performed with a gem 561 laser (Laser Quantum, Stockport, UK) or a 785 nm laser (iBeam smart WS CW Laser). 2D excitation emission spectra were collected in the same setup as above with a Monochromator MSH150, equipped with a LSE341 light source (LOT-Quantum Design GmbH, Darmstadt, Germany) as excitation source.
NIR fluorescence analyte response measurements were performed, by using 180 µL of a 0.2 nM ssDNA-SWCNT solution (calculation of molar nanotube concentration based on an approach by Schöppler et al.[3]) or 180 µL of a 0.1 absorbance (measured at E_{11} transition, ~ 995 nm) PEG-PL-SWCNT solution. NIR fluorescence spectra were acquired at 150 mW excitation (561 nm) and 3 s integration time and 200 mW (785 nm) and 10 s integration time. Dose-response measurements were fitted with a one site – specific binding fit or with a hyperbolic fit (GraphPad Prism 9) using $Y = B_{max} * X / (K_d + X)$ with X = concentration of the analyte; Y = specific binding; B_{max} = Maximum binding and K_d = dissociation constant.

SWCNT separation

Separation of (6,5)-SWCNTs was performed according to a previously reported aqueous two-phase extraction (ATPE) protocol from Li *et al*.[4] Briefly, in a three step approach SWCNT chiralities were separated between two aqueous phases, containing dextran (MW 70000 Da, 4% m/m) and PEG (MW 6000 Da, 8% m/m) through varying pH-values *via* HCl addition. The final B3 (bottom)-phase yielded monochiral (6,5)-SWCNTs, which were dialyzed against a 1% (w/v) DOC solution. After further surfactant exchange to sodium cholate, a similar dialysis approach for PEG-PL was performed as described before.[2,5] Separation of (7,6)-SWCNTs and their surface modification to ssDNA was performed following a recently published protocol.[5]

NIR stand-off imaging

NIR stand-off detection was performed with a custom made, portable set-up[6], using a XEVA (Xenics, Leuven Belgium) NIR InGaAs camera (Kowa objective, f = 25 mm/F1.4) with a 900 nm long pass filter (FEL0900, ThorLabs) and a white light source (UHPLCC-01, UHP-LED-white, Prizmatix) equipped with a 700 nm short pass filter (FESH0700, ThorLabs) for excitation. Stand-off distance for NIR fluorescence detection for the seedling-agar experiments (3 s integration time, light intensity 48 $mWcm^{-2}$) was 20 cm. Light intensity was measured at 570 nm with a power meter (PM16-121, ThorLabs). Hyperspectral imaging was performed with additional 950 nm (FEL0950, ThorLabs) and 1100 nm (FEL1100, ThorLabs) long pass filters and 5 s integration time.

Plant material and classical polyphenol detection
<u>Plants and Insects</u>

Tococa guianensis plants were raised from cuttings in a glasshouse (day, 23-25°C; night, 16-18°C; 60-70% rel. humidity; 16 h/8 h light/dark cycle). Once the plants were big enough, they were potted into a 400 mL pot filled with a mixture of 1/4 Klasmann potting substrate (Klasmann-Deilmann, Geeste, Germany), 1/4 Latvian white peat, 1/4 pine bark (7-15 mm), 1/8 sand and 1/8 Legan (5-7 mm) and inoculated with BioMyc™ Vital Mykorrhiza (BioMyc, Brandenburg/Havel, Germany). Experiments were performed with one-year-old Tococa plants.

Field studies on *Tococa quadrialata* were conducted in the Tambopata Reserve [12° 50' 10'' S, 69° 17' 34 '' W] close to the Explorer`s Inn lodge in the lowland Amazon basin in Peru at an elevation a.s.l. 210 m. The average annual rainfall is 2335 mm with a dry season from June until October. The maximum monthly temperature is around 30° C whereas the monthly minimum is around 19 °C. The subpopulation of *Tococa quadrialata* used for this study was found alongside a small path. All these myrmecophytic plants were colonized by ants of the genus *Azteca spec.* (subfamily Dolichoderinae). The experiment was performed and leaves were sampled when the plants were on average 56 cm tall and had 14 leaves.

Spodoptera littoralis larvae used for the greenhouse herbivore experiment were hatched from eggs and reared on an agar-based optimal diet at 23°C–25°C with 8 h light/16 h dark cycles.[7] Third instar *S. littoralis* larvae were chosen for the herbivory experiment and starved 24 h prior to plant feeding. For the field experiments, *Spodoptera* larvae of third to fifth instar were collected from a local area.

<u>Herbivore treatment</u>

One leaf of the second pair of fully expanded leaves was enclosed with a perforated PET bag. Two *Spodoptera* larvae were released on the bagged leaves and allowed to feed for 24 h, whereas the control leaves were enclosed with an empty bag for 24 h. At the end of the experiment, these leaves were excised and photographed to determine the leaf damage, domatia were removed and the leaves flash-frozen in liquid nitrogen. The field samples were lyophilized for the transport to Germany and subsequent chemical analyses.

<u>Cell culture of *Glycine max*</u>

Soybean (*Glycine max* L. cv. Harosoy 63) cell suspension cultures were cultivated according to Fliegmann et al.[8] Briefly, cells were kept in the dark at 26 °C under shaking conditions (110 rpm) and were subcultured in fresh medium every 7 days. To document the accumulation of phenolic compounds with age, subcultures were harvested after 2, 7 and 14 days. More specifically, the cultures were filtrated utilizing Whatman® Grad 1 filter paper (GE Healthcare, Chicago, IL, USA) and the filtrates were used for further analyses.

For the induction of isoflavonoid production, soybean cell suspension cultures were sub-cultured after 5 days in fresh medium (6 g fresh mass per 40 ml medium) and after another 2 days of growth, 1 mL of suspension culture was carefully transferred to each well of a 24 well plate **(**CELLSTAR® 662102, Greiner Bio-One, Kremsmünster, Austria). 10 µL of 50 mg/mL raw elicitor isolated from the cell walls of the phytopathogenic oomycete *Phytophthora sojae* in ddH_2O was added to half of the wells. Wells to which 10 µL of pure ddH_2O were added served as control treatment. The plate was kept in the dark at room temperature under constant shaking conditions (100 rpm) for 4 days. The suspensions were then carefully transferred to 1.5 mL Eppendorf tubes (Eppendorf, Hamburg, Germany), cells spinned down (5000 rpm, 4 °C, 20 min) and the supernatant transferred to a new vial for subsequent chemical analysis. As a negative control, six 1 mL samples of the starting culture were transferred to 1.5 mL Eppendorf tubes instead of the well plate and harvested immediately. 20 µL of the cell suspension supernatant was directly added to the nanosensors for polyphenol detection.

<u>Extraction of ellagitannins and anthocyanins from *Tococa*</u>

1 g freeze-dried, finely ground powder of *Tococa* leaves was extracted five times with 10 mL of acetone/water (7/3, v/v) containing 0.1 % (m/v) ascorbic acid to obtain the crude extract. Acetone was evaporated below 40 °C with a rotary evaporator and the remaining water phase was subjected to SPE using a CHROMABOND® HR-X column (6 mL, 500 mg; Macherey-Nagel, Düren, Germany). After the equilibration of the column with methanol and water, the extract was applied to the column and different fractions were subsequently eluted with water, increasing concentrations of acetone/water (1/1, 3/1, *v*/*v*) and acetone. Fraction volume was 6 mL per eluent, whereas three fractions per one eluent were collected. Whenever it was possible to obtain smaller fractions of similar chemical compounds based on visual hints, fractions smaller than 6 mL were collected. Fractions were analyzed by a diode array detector (DAD) system after separation by Agilent 1100 HPLC (Agilent Technologies, Santa Clara, CA, USA) using a Luna® Phenyl-Hexyl 100 Å (4.6 × 150 mm, 5 µm, Phenomenex, Aschaffenburg, Germany) column. The binary mobile phase consisted of acetonitrile (A) and 0.5 % trifluoroacetic acid in water (B) at the flow rate of 1 mL min^{-1}. The elution profile was: 0 – 18 min, 5 – 41 % A in B; 18 – 18.1 min, 41 – 100 % A in B; 18.1 – 21 min 100 % A in B; 21 – 21.1 min 100 – 5 % A in B; 22 – 26 min 5 % A. Absorbance was detected at 254 nm, 270 nm, 280 nm and 520 nm. 1 mL of the fraction of interest was dried under nitrogen stream to yield 38 mg of a red glittering solid residue enriched in ellagitannins and anthocyanins. This fraction was dissolved in MeOH and diluted to the desired concentration. 2 µL of this solution was added to the nanosensor solution for polyphenol sensing.

<u>Further polyphenolic compounds</u>

Anthocyanidins used for nanosensor screening were extracted from dried hibiscus (purchased in a local store), following a described protocol.[9] Trihydroxypterocarpan (THP) were extracted from soybean according to previous literature.[10,11]

<u>Targeted analysis of herbivore treated leaves</u>

40 mg of freeze-dried ground leaf powder or 100 mg of frozen fresh leaf powder ground in liquid nitrogen were extracted with 1 mL of methanol (MeOH) containing 10 ng/mL trifluoromethyl-cinnamic acid as an internal standard. The homogenate was mixed for 30 min and centrifuged at 16000 rcf for 10 min. The supernatant was used for subsequent analysis. Compound separation and targeted analysis was achieved by LC-MS/MS as described in Lackus et al.[12], using multiple reaction monitoring to monitor analyte parent ion → product ion formation for detection of the phenolic compounds (Catechin: *m*/*z* 289→109; DP: -30 V CE: -32 V; Apigenin: *m*/*z* 269→117, DP: -30 V, CE: -44 V; TMCA (internal standard): *m*/*z* 215 → 171 DP: - 30 V, CE: -18 V).

Chromatograms were analyzed using the software Analyst 1.6.3 (Applied Biosystems) with automated peak integration. The internal standard was used to normalize the peak areas. 2 µL of these MeOH extracts were added to the nanosensor solution for polyphenol sensing.

High-resolution mass spectrometry of *Tococa* and soybean samples

The methanolic extracts and interesting fractions of *Tococa* and the aqueous supernatants of the soybean cell cultures were furthermore subjected to high-resolution mass spectrometry in order to obtain the accurate masses of the compounds. Samples were analyzed with a Dionex Ultimate 3000 RS Pump system (Agilent) coupled to a timsTOF mass spectrometer with a turbospray ion source (Bruker Daltonics, Bremen, Germany). Separation was achieved as described by Lackus et al.[12] The mass spectrometer was successively operated in negative and positive ionization mode scanning a mass range from *m*/*z* 50 to 1,500 with the capillary voltage set at 4,500 V (positive mode) or 3500 V (negative mode), respectively. Nitrogen served as drying gas (8 L/min, 280°C) and nebulizer gas (2.8 bar). As internal calibrators, sodium formate adducts were used. Compass Hystar 3.2 (Bruker) was used for data acquisition and MetaboScape (Bruker) for data processing, sum formula calculation, structure prediction and preliminary statistical analysis. Additionally, SciFinder (https://scifinder.cas.org) was used for structure prediction and compound identification. Peak integration of extracted ion chromatograms corresponding to known soybean compounds was achieved with Compass Quant Analysis (Bruker).

Total phenol content quantification

An established protocol[13] enables the colorimetric quantification of the total phenol content. In short, 100 µL sample were mixed with 200 µL Folin–Ciocalteu reagent (10 %; *v*/*v*) and 800 µL Na_2CO_3 (700 mM) and left for reaction (1.5 h at RT). Reaction tubes were shortly centrifuged (2 min @ 16.100x *g*) and subsequently the absorbance of the solution was measured at 765 nm with a JASCO V-670 device. Soybean cultures were directly used, while *Tococa* extracts were diluted accordingly, to not exceed the linear range of the calibration.

Incorporation of *G. max* seedlings into NIR fluorescent agar

PEG-PL-SWCNTs sensors were incorporated into 5 mL 0.4 % agarose culture medium (containing Murashige & Skoog (MS) media) and casted into a sterile petri dish (Ø 8.5 cm), yielding a final SWCNT absorbance of 0.3 at the E_{11} transition at ~995 nm. Further UV sterilization was performed for 15 min (UV-Kontaktlampe Chroma41, 254 nm, Vetter GmbH) before placing 3-days old seedlings of *Glycine max* L. cv. Maraquise or cv. Edamame Green Shell onto the sensor-agar. The root of the seedling was covered with 10 mL 0.8 % agarose MS medium. Polyphenol sensing experiments were performed after >12 h post seedling incorporation.

Polyphenol visualization

Seedlings were challenged with 50 µL (10 mg/mL) raw-elicitor of *Phytophthora sojae*, or as a control 50 µL ddH_2O, after wounding the root tissue with a 0.4 mm cannula. Automated image recoding was performed for every 30 min over 10 h and subsequently after 24 h, using 3 s integration time. Excitation was synchronized in the same way, whereby only for NIR fluorescence acquisition the illumination system was turned on for 2 min.

Image analysis

NIR images were acquired with Xeneth Software 2.7 (Xenics, Leuven Belgium) and converted in ImageJ (1.51k) into 8-bit data format. Differential NIR fluorescent images (I/I_0) were obtained by dividing the time series of the recorded images (I) by the first image (I_0), while using a masked root tissue region. The intensity changes in the 32-bit images were then further analyzed with a 5-pixel with line profile or using a 500-pixel large area, close to the challenged root position.

Hyperspectral images were analyzed by subtracting the corresponding 1100 nm long pass filter images from the ones acquired with a 900 or 950 nm long pass filter, to obtain the fluorescence intensities of the (6,5)-SWCNTs which emit at ~ 1000 nm. Fluorescence emission of (7,6)-SWCNTs were detected with a > 1100 nm long pass filter.

Results and Discussion

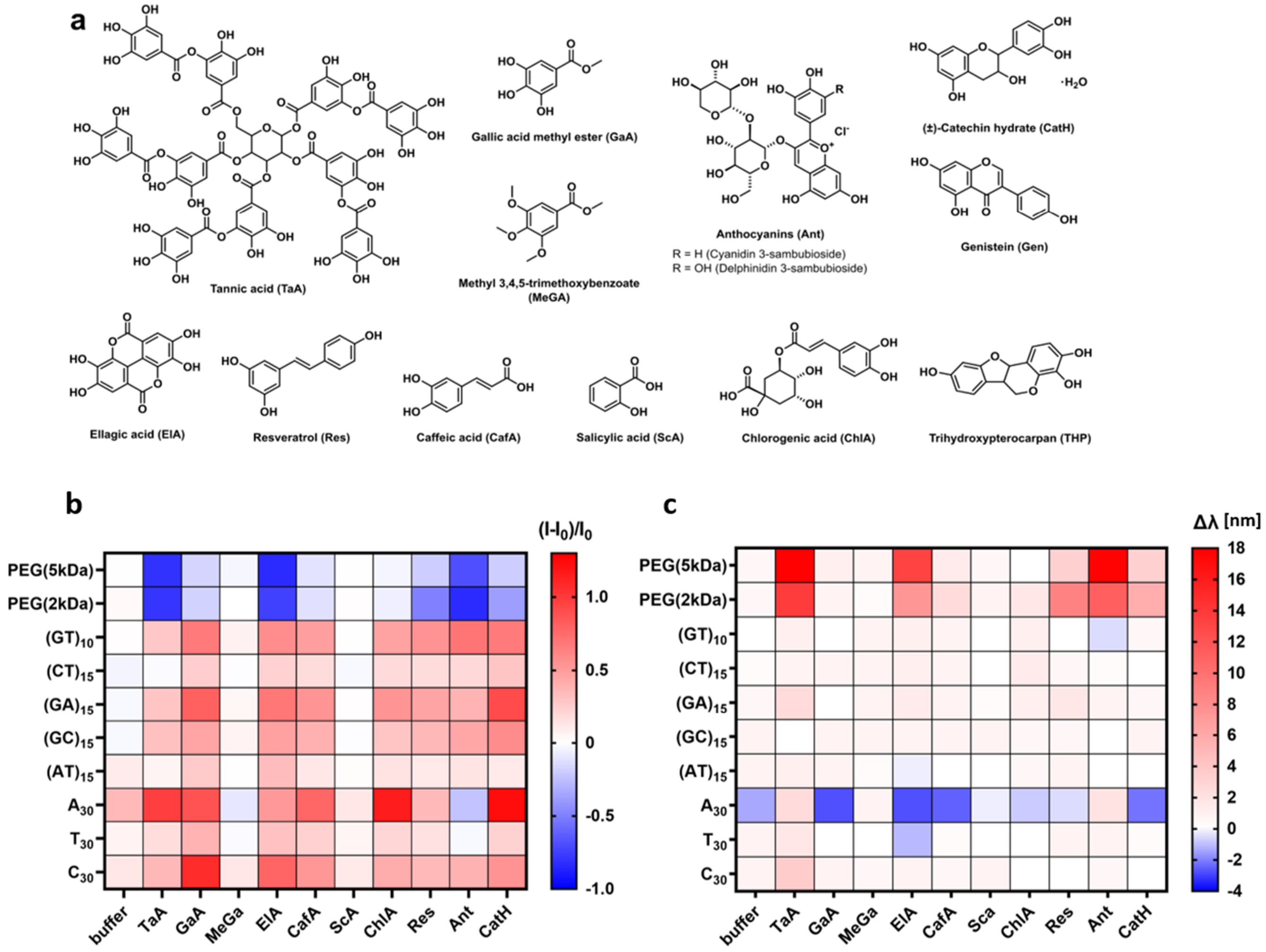

Figure S1: Sensor screening for plant polyphenol detection.

a) Chemical structures of the used polyphenolic compounds. b) Fluorescence changes ($I-I_0/I_0$) of all SWCNT-based sensors with different surface modifications, in response to different plant polyphenols summarized in a heatmap (mean, n = 3). Shades of blue indicate fluorescence decrease and shades of red fluorescence increase, within the indicated range. c) Emission wavelength changes (*Δ*λ) of all SWCNT sensors after polyphenol addition. Shades of blue indicate hypsochromic shift and shades of red bathochromic shift, within the indicated range (polyphenol concentration = 10 µM; TaA = 1 µM).

ssDNA-SWCNTs responded with a general pattern of fluorescence increase, however, showing distinct differences in sensing magnitudes. PEG-PL-SWCNTs on the other side responded with a fluorescence decrease. Note: It is known, that A-rich ssDNA-SWCNTs exhibit decreased colloidal stability over time[5], which affects aggregation/quenching state, biassing the total emission intensity and sensors response. Moreover, a variety of molecules can modulate their photoluminescence, which makes this particular ssDNA modification less suitable for further sensor design. For the sake of completeness, we showed these screening results, but furthermore excluded A_{30}-SWCNTs for final polyphenol sensor development. To exclude pH or ionic strengths related sensing effects[14], all experiments were performed under buffered conditions. $(AT)_{15}$-ssDNA surface modification seem less sensitive to the tested polyphenolic compounds, which would make them a suitable reference material for multichiral sensing.

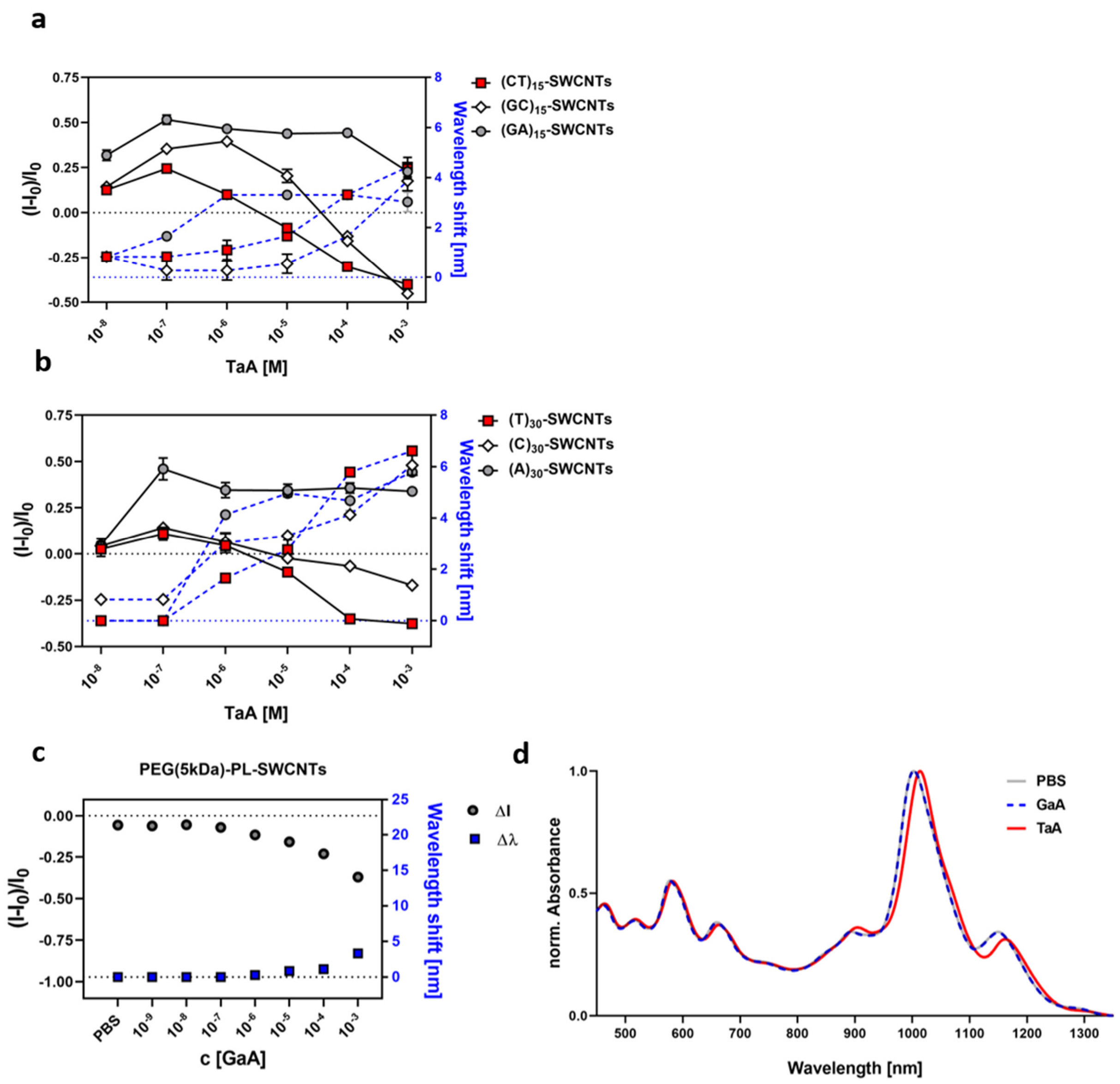

Figure S2: Sensing of tannic acid (TaA).

a) and b) Concentration dependent NIR fluorescence shifts of multiple ssDNA-SWCNTs in response to tannic acid. Intensity (black line) increases in the nM range, while higher concentrations lead to decreased fluorescence emissions, whereas wavelengths shift (dotted blue line) increases with TaA concentration (mean ± SD, n = 3). This observation could be the result of two different principles of interaction. The increase could be similar to the one known for dihydroxy group containing catecholamines[3] (like dopamine), so polyphenols could push the DNA-phosphate backbone closer to the SWCNT surface. At higher concentrations, polyphenol could lead to colloidal instability of the polymer-SWCNT complex, as known for PEG or protein precipitations[4], resulting in the fluorescence decrease. Since the fluorescence modulation from ssDNA-SWCNTs (e.g. $(CT)_{15}$- and $(GA)_{15}$-SWCNTs) were observed with quite different magnitudes, these types of interface modifications were excluded as further polyphenol-sensitive probes. c) GaA addition only slightly alternates the emission of PEG-PL-SWCNTs (mean ± SD, n = 3), indicating that the three-dimensional architecture of structurally large polyphenols contributes significantly to the sensing mechanism. d) TaA interaction with PEG(5kDa)-PL-SWCNTs causes a ~10 nm bathochromic shift in E_{11} absorbance maximum, while GaA does not cause a spectral shift (TaA, GaA = 10 µM). The absorption at the excitation wavelength (E_{22} transition), however, just showed a minor shift. Therefore, the observed shifts at the E_{11} transition could correlate partly with the detected energy differences in fluorescence emission. However, we assume, that interaction of PEG-PL-SWCNTs with certain polyphenols will increase the local dialectic constant around the SWCNTs, thus causing the red-shifted emission features.

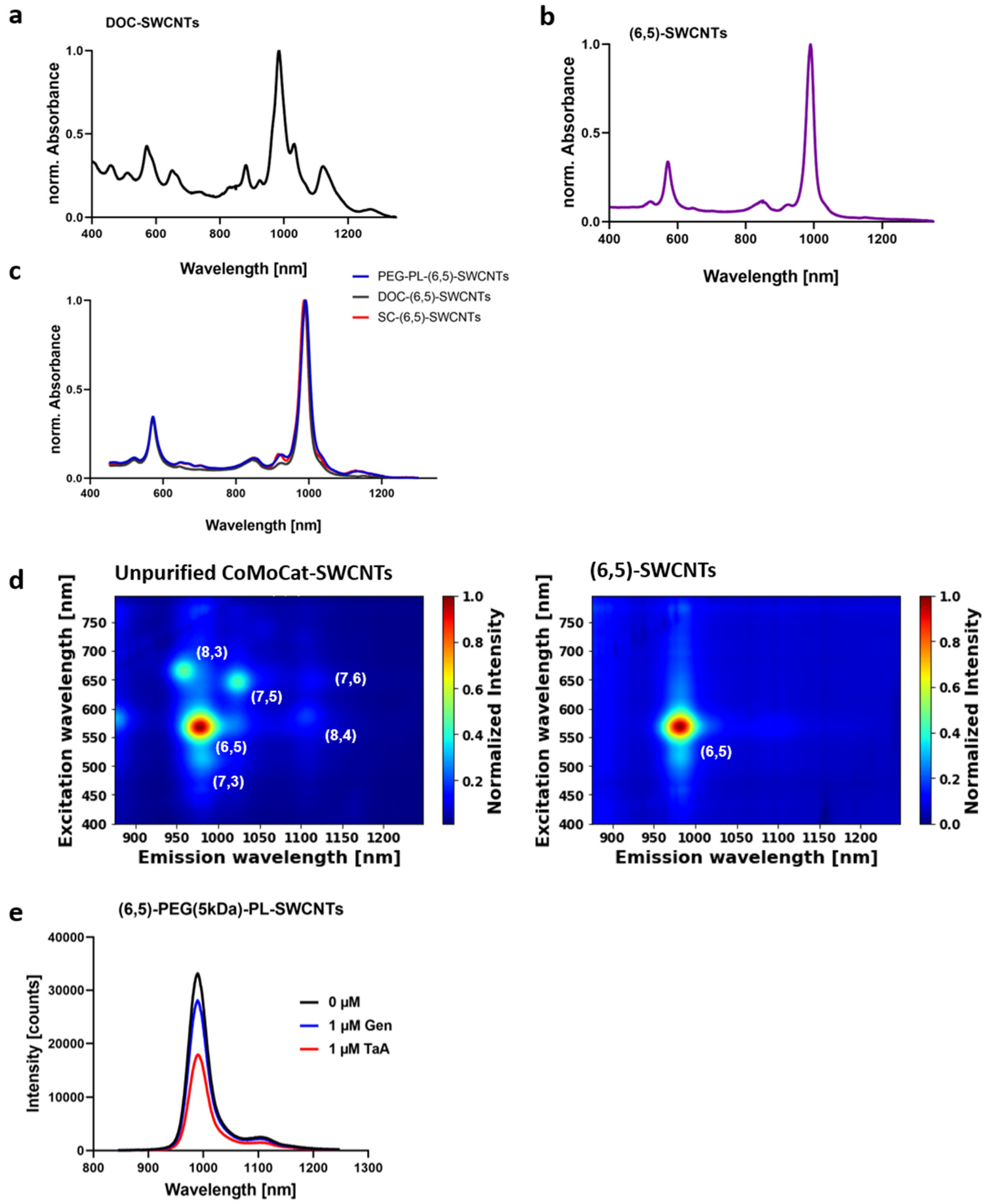

Figure S3: SWCNT purification to obtain monochiral PEG-PL-(6,5)-SWCNT sensors.

a) Normalized absorbance spectra of unpurified CoMoCat-SWCNTs dispersed in 1% DOC. b) Normalized absorbance spectra of purified (6,5)-SWCNTs, obtained by multiple step aqueous two-phase extraction (ATPE), following an approach described by Li et al.[4] c) Normalized absorbance spectra of (6,5)-SWCNTs, exchanging the surface modification from sodium deoxycholate (DOC) to sodium cholate (SC) and to a biocompatible polyethylenglycol-phospholipid (PEG-PL). d) Corresponding 2D photoluminescence spectra of the non-purified and the purified (6,5)-SWCNTs. e) Exemplaric fluorescence spectra of monochiral PEG(5kDa)-PL-(6,5)-SWCNTs before and after addition of tannic acid and genistein, showing a strong fluorescence decrease.

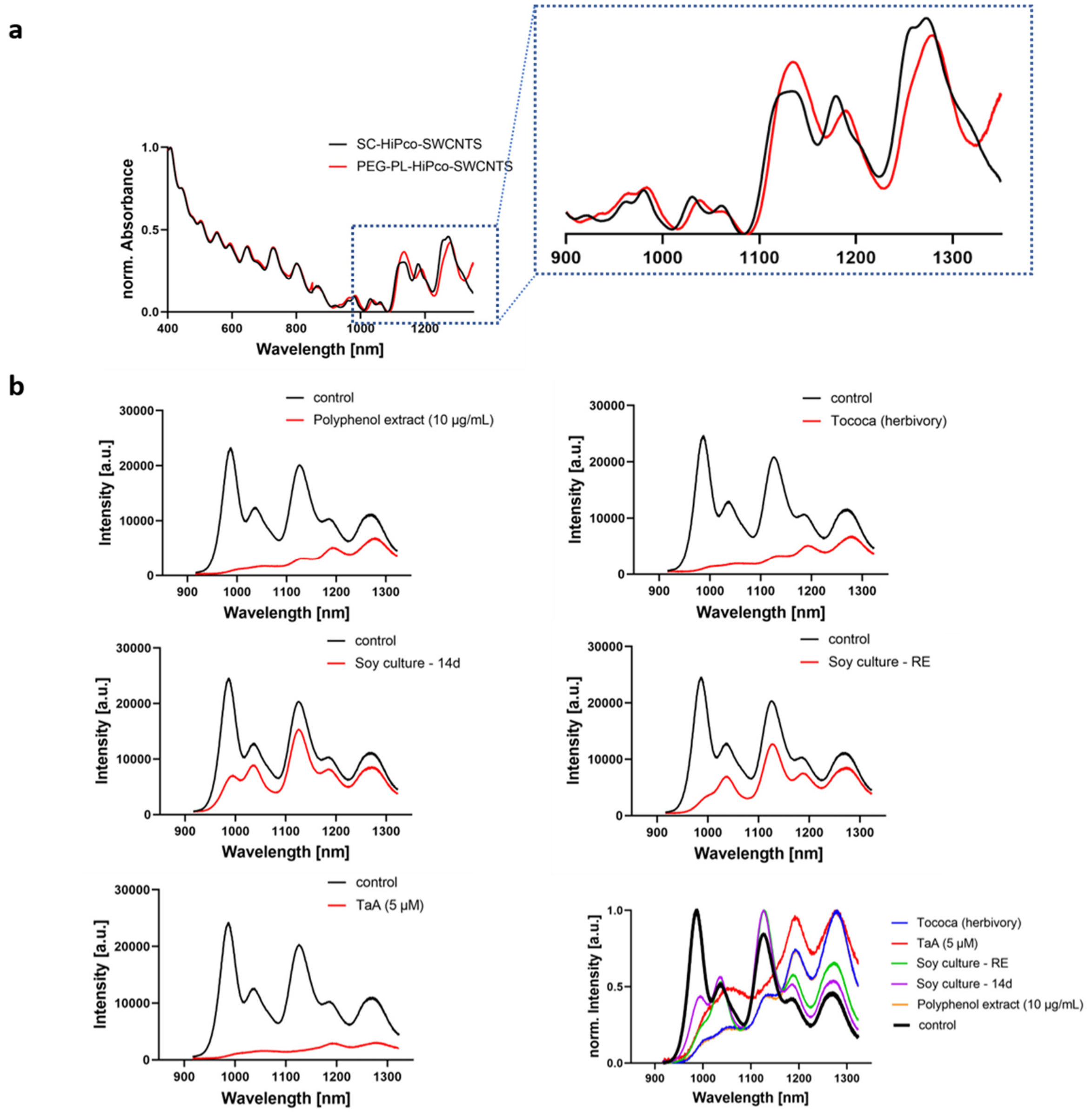

Figure S4: Polyphenol interaction with HiPco-SWCNTs.

a) Normalized absorbance spectra of SWCNTs dispersed in SC and after further surface exchange to PEG-PL. Additionally, a magnified E_{11} transition region is shown. b) NIR fluorescence spectra of PEG-PL-HiPco-SWCNTs before (black spectra) and after (red spectra) the addition of polyphenol containing plant extracts or TaA (*Tococa* polyphenol extract and MeOH extract from herbivory treated plants). Strong photoluminescence modulations are visible with certain differences between SWCNT chiralities, which could point towards a chirality dependent effect regarding the polyphenol composition (see last plot, compared as normalized fluorescence emissions).

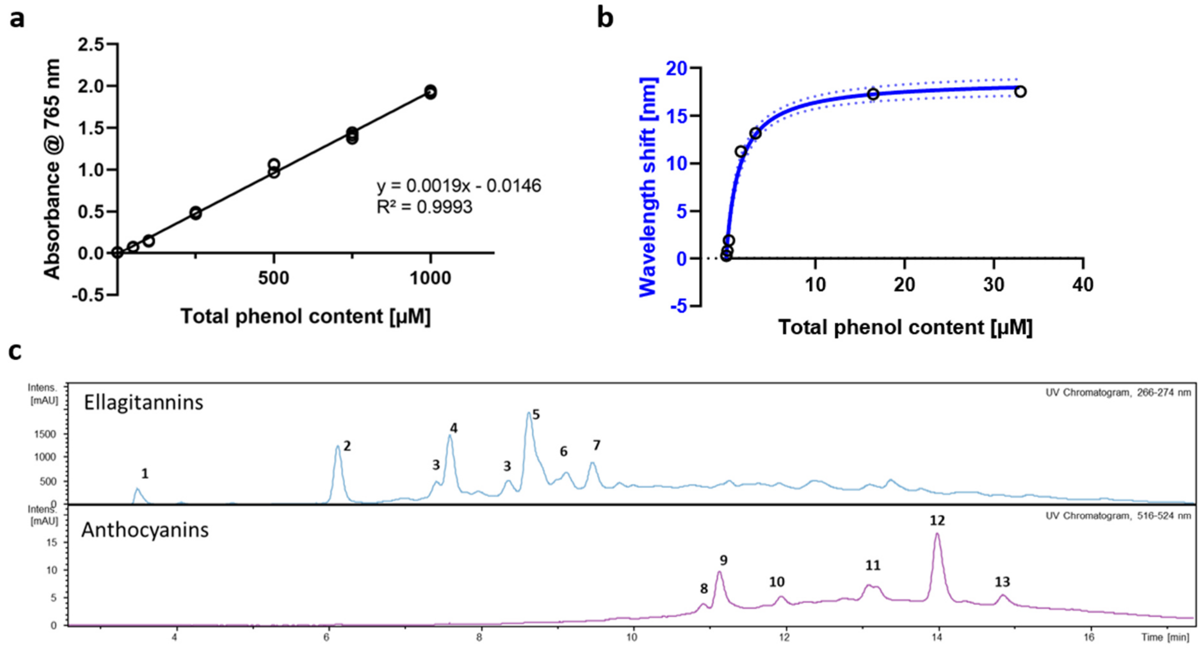

1-7	Ellagitannins
8-12	Anthocyanins

Figure S5: Total phenol content quantification and analysis of *Tococa spp.* polyphenol extract.

a) Calibration curve (linear regression) for the colorimetric assay[13] used to assess the total phenol content. Known concentrations of gallic acid are challenged with the Folin–Ciocalteu reagent, while quantifying the absorbance at 765 nm (n = 3). The resulting calibration, expressed as gallic acid equivalents, shows a linear trend in the µM regime. b) Corresponding plot similar to Figure 3c. It shows the PEG-PL-SWCNT response to purified polyphenol extract from *Tococa spp*., expressed as the total phenol content vs. wavelengths emission shifts (mean ± SD, n = 3, blue line = hyperbolic fit). Compared to the classical Folin–Ciocalteu assay, the sensors display a highly dynamic response in the low µM range with a K_d of 1.5 µM. c) HPLC-UV-Vis chromatogram of the used *Tococa spp.* sample, containing all extractable leaf polyphenols with a predominantly high ellagitannin content, as seen from the counts in the UV trace (266 -274 nm). Substance assignment as ellagitannins and anthocyanins were further confirmed by high resolution mass spectrometry and comparison of the LC-MS results to literature.[15–17] As the sensors probe the total phenol content and the response depends partwise on the polyphenol profile, it would be likely most accurate to create a sensor calibration curve for each plant species/genus of interest.

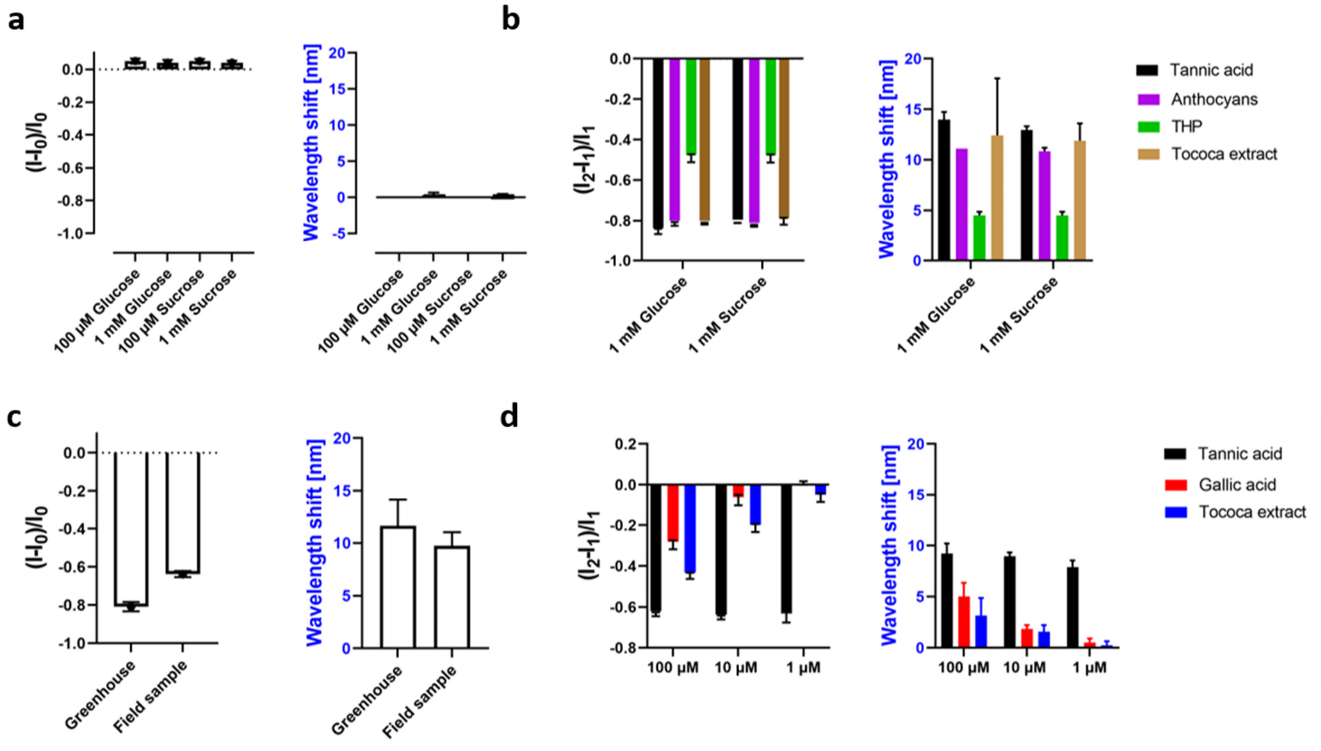

Figure S6: Polyphenol sensing with sugar and leaf extract background.

a) Polyphenol sensing with mono- and disaccharide background. Addition (I) of up to 1 mM glucose or sucrose is not changing the nanosensors responses. b) Further addition (I_2) of polyphenols leads to a similar sensor response, as seen in previous experiments in PBS (10 µM polyphenol concentration, 10 µg/ml extracted polyphenol fraction for the Tococa extract (similar to Figure 3c and S5), mean ± SD, n = 3). However, it should be mentioned, that the intensity changes of the sensors were unbiased, compared to similar experiments in the absence of saccharides, while the detected shift in emission wavelength was slightly reduced (e.g. Tococa extract shifted nanosensor emission by 17 nm w/o saccharides and ~12 nm with glucose/sucrose background). Since 2 µL leaf extract is added to 180 µL sensor solution, such unphysiological high background concentrations of sugars would not be expected in primary samples. c) Polyphenol sensing with a Tococa leaf extract background. Addition (I) of 2 µL Tococa leaf extract, ether from a 'field' or 'greenhouse sample' (see materials and methods), leads to a strong sensor response. d) Further addition (I_2) of polyphenols to e.g. 'field sample' can still be sensed in a concentration dependent way, illustration the principle of a standard addition in a chlorophyll (and polyphenol) containing background (mean ± SD, n = 3). Similar results were obtained, when spiking the 'greenhouse sample', showing slightly smaller responses. Note that wavelengths shifts in (d) are compared to the addition (I) of totoca extracts, so overall shifts to the starting conditions (I_0) would be e.g. for tannic acid close to 20 nm.

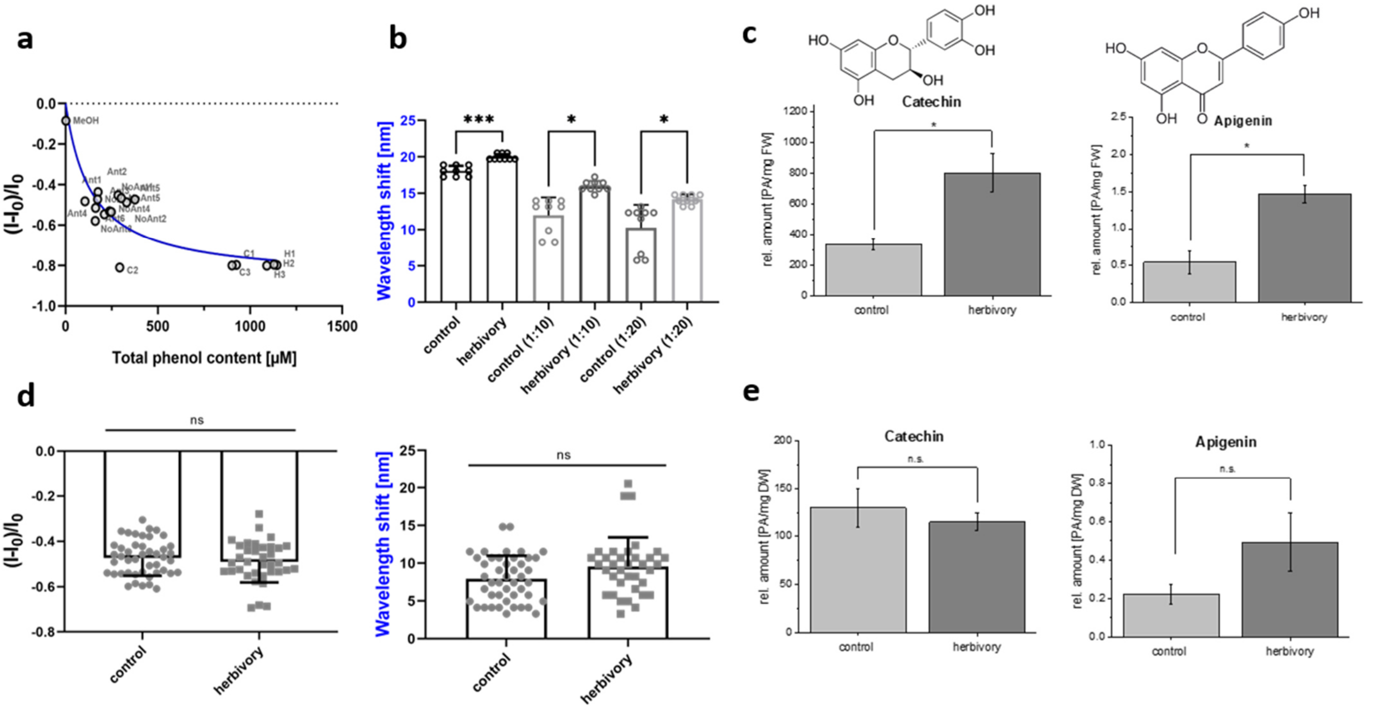

Figure S7: Analysis of crude *Tococa spp.* leaf extracts.

a) Correlation of the intensity changes against the total phenol content from multiple *Tococa* leaf MeOH-extracts (corresponding plot to Figure 3d, 2 µL undiluted MeOH extract was added to the nanosensors). It should be mentioned, that first unknown Tococa extracts were analyzed by the nanosensors and then correlated with the established Folin–Ciocalteu assay. For such a species-specific calibration fit, a dynamic response in the µM range of total phenols is observed (expressed as gallic acid equivalents) (mean ± SD, N =1, n = 3, blue line = hyperbolic fit). b) When diluting the plant extract from herbivore treated *Tococa* plants from the greenhouse, the phenol concentration is shifted to the dynamic response region of the nanosensors. This increases the mean difference in emission wavelength shift, which is hereafter still significantly different (complementary to Figure 3e, mean ± SD, N =3, n = 3). c) HPLC-MS quantification of catechin and apigenin as two prominent examples show as well a significant increase after herbivore treatment. d) Nanosensor responses of different extracts from wild *Tococa* plants show no significant difference in polyphenol levels after herbivore attack, which was carried out as described in the MM section. Hereby, a strong variation in the total phenol concentration is detected, pointing towards more complex interactions in the wild plants (e.g. previous pathogen / herbivore attack mediated a constant increase in polyphenol levels) (mean ± SD, N =6, n = 3). e) HPLC-MS quantification confirmed those results, by showing no significant differences in the most prominent flavonoid compounds (note that apigenin levels show a slight increase, not effecting the overall total phenol concentration, as seen from magnitudes higher, not varying catechin levels).
(nanosensor analysis: ***P<0.001; *P<0.03; ns = not significant; unpaired t-test; PA = peak area, FW = fresh weight, DW = dry weight)

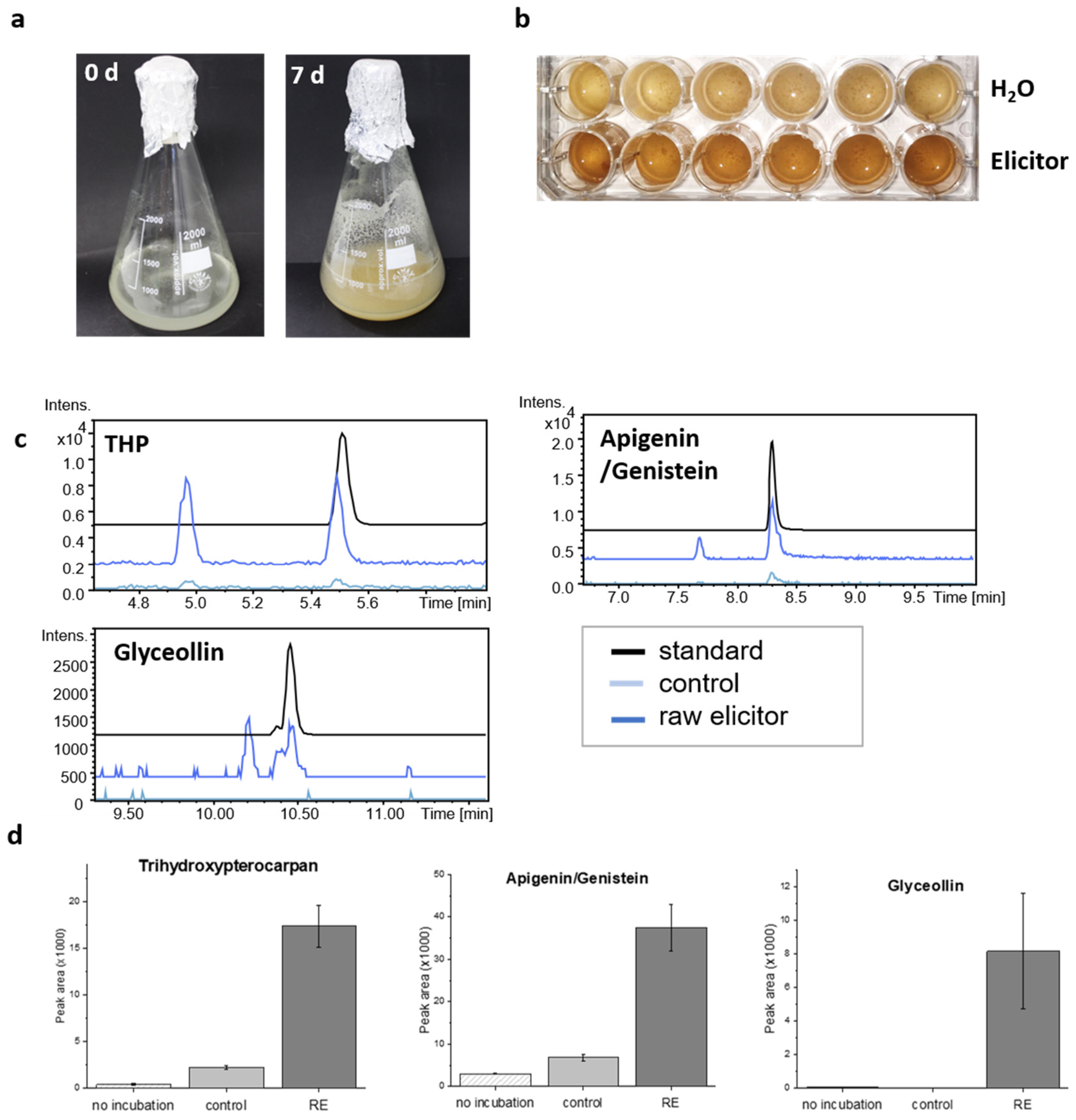

Figure S8: Characterization of polyphenols from soybean cell cultures.

a) Pictures of the soybean cultures show a clear browning after 7 days, indicating a release of polyphenols during growth and maturation. b) Six soybean culture replicates, stimulated with 0.5 mg/mL raw elicitor (lower row) or with H_2O (upper row) as a control. Again, a clear browning of the induced cultures is visible, which indicates accumulation of phenolic compounds.[18] c) Extracted ion chromatograms (EIC) of the most abundant *m*/*z* values of the respective standards are shown. Comparison of these signals in samples from raw elicitor (RE) treated and control cultures shows that these compounds are accumulating after stimulation with RE. d) Peak areas of these EIC signals were used for relative quantification of the compounds. A drastic increase in flavonoids after raw elicitor treatment was detected.

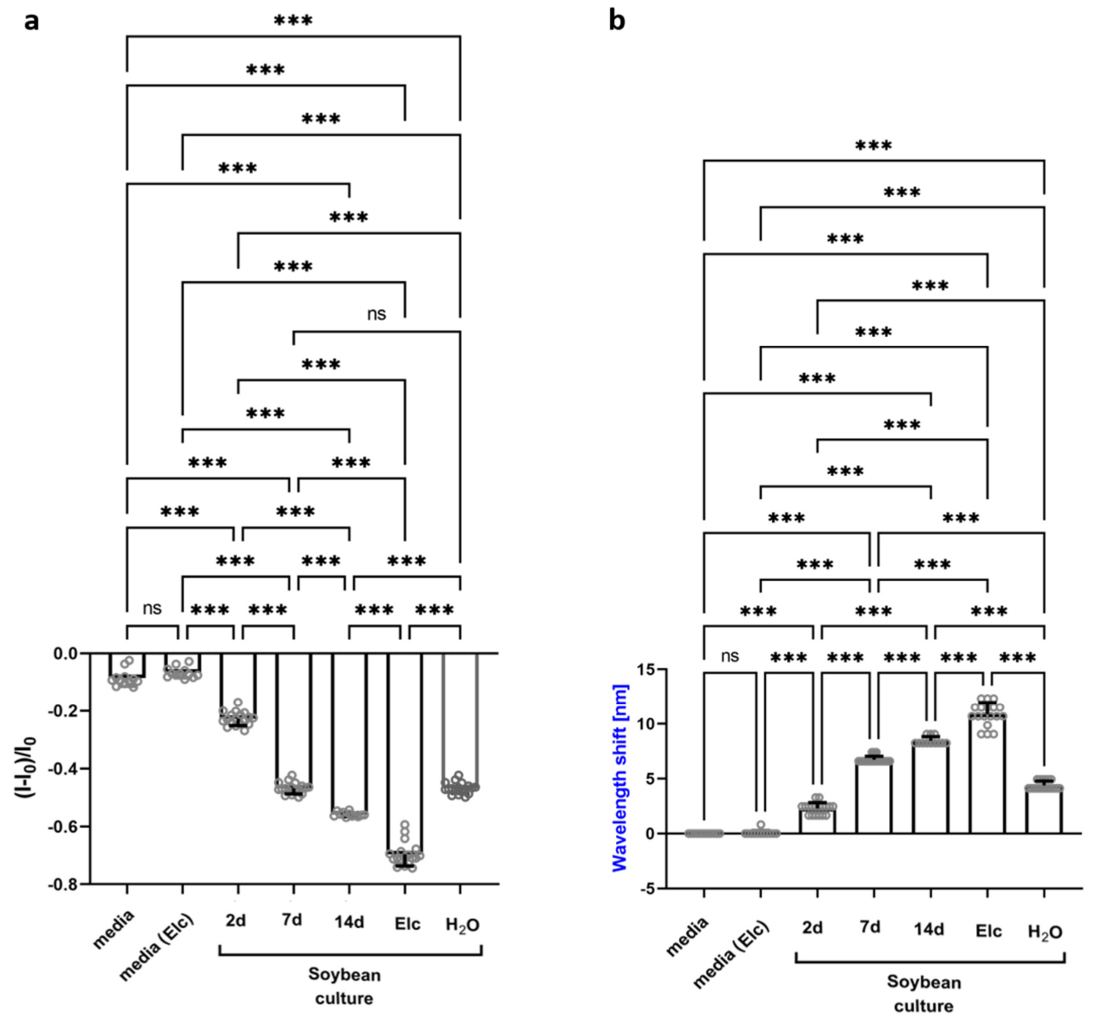

Figure S9: Sensor response to soybean cell cultures.

a) and b) Full statistical analysis (one-way ANOVA) for the shown sensor responses in Figure 3f and 3g. Here, soybean cell cultures (as cell-free supernatants) are added to the PEG-PL-SWCNT nanosensors, which react with a fluorescence decrease and emission wavelengths shift to the age and stimulus (elicitor, Elc) dependent polyphenol content (mean ± SD, N = 6, n =3; nanosensor analysis: ***P<0.001; ns = not significant).

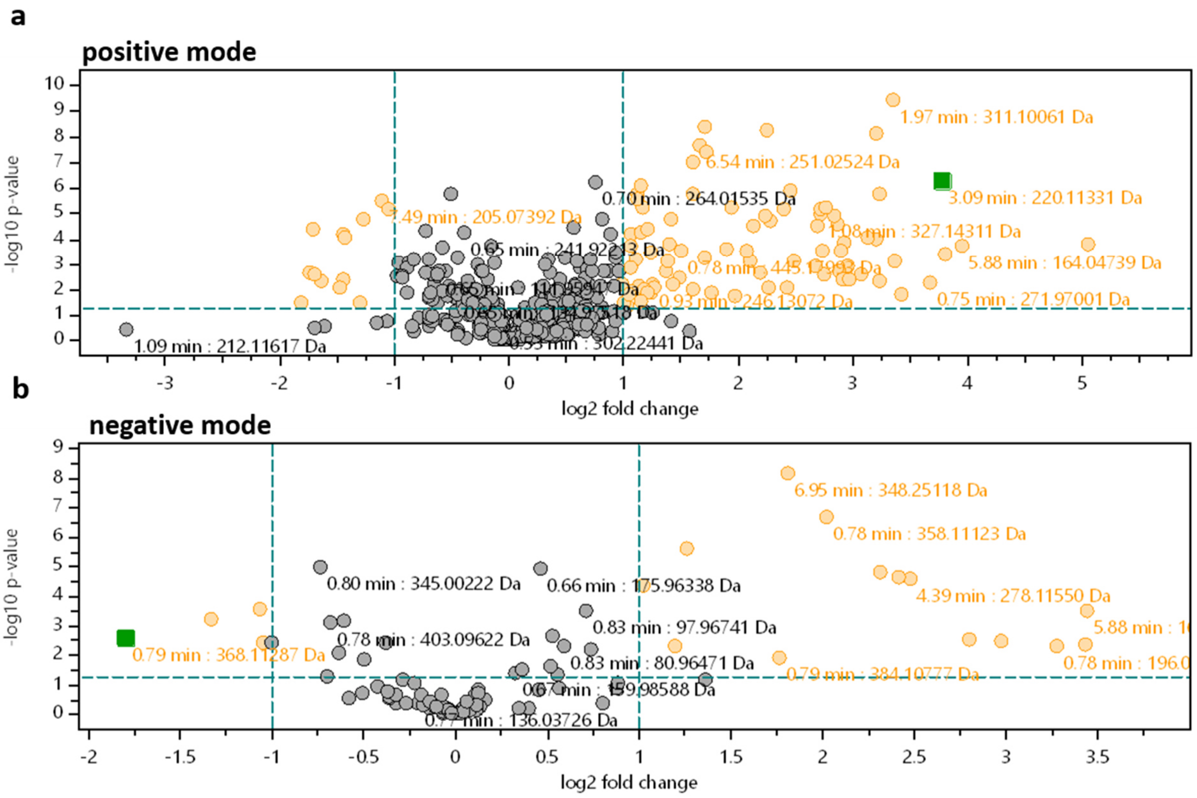

Figure S10: Volcano plot comparing feature abundance in control and elicitor induced soybean cell cultures.

LC-MS analysis of control and elicitor induced soybean cell cultures was conducted with the mass spectrometer operating in positive (a) and negative (b) ionization mode to compare the relative abundance of the extracted features. The volcano plot visualizes the metabolic differences (orange features, $p<0.05$, fold change > 2) and non-affected features (grey) upon raw elicitor treatment. Green color marks the selected feature, which is not relevant for this result.

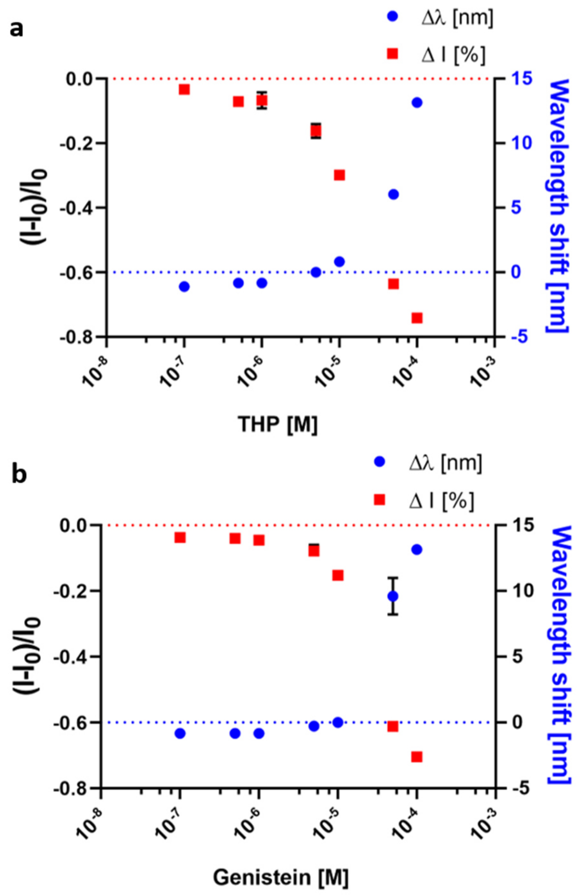

Figure S11: Sensor response to prominent soybean polyphenols.

a) Trihydroxypterocarpan (THP), an important compound released after pathogen (elicitor) stimulus and b) genistein, which is released during maturation, quench and shift the nanosensor fluorescence in a similar concentration dependent manner (mean ± SD, n =3).

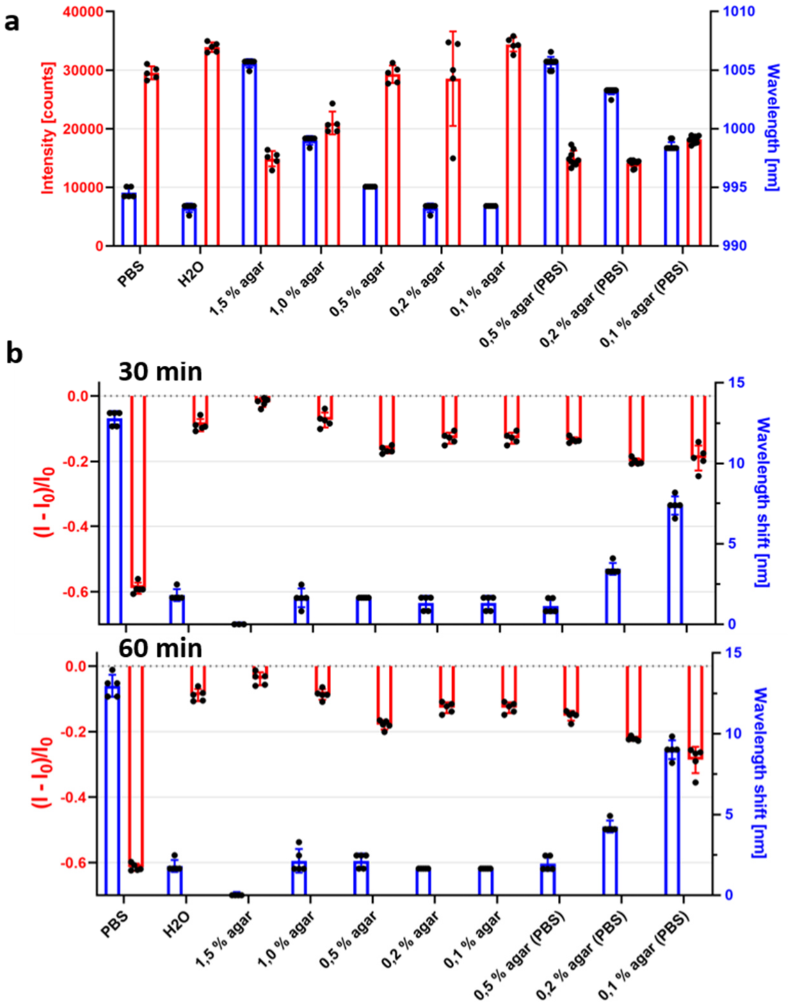

Figure S12: Tuning the nanosensor incorporation into functional agar medium.

a) Photoluminescence properties (intensity and emission wavelength) of PEG-PL-SWCNTs in different environments. 90 µL SWCNT solution was placed in a 96-well plate and analyzed. Increasing agar concentration decreases the emission intensity and redshifts the maxima (mean ± SD, n = 5). b) Sensing of polyphenols, here genistein with a final concentration of 100 µM, seems to be strongly affected by a higher agar concentration, as well as by the ion concentration (agar system in H_2O or in PBS) (mean ± SD, n = 5).

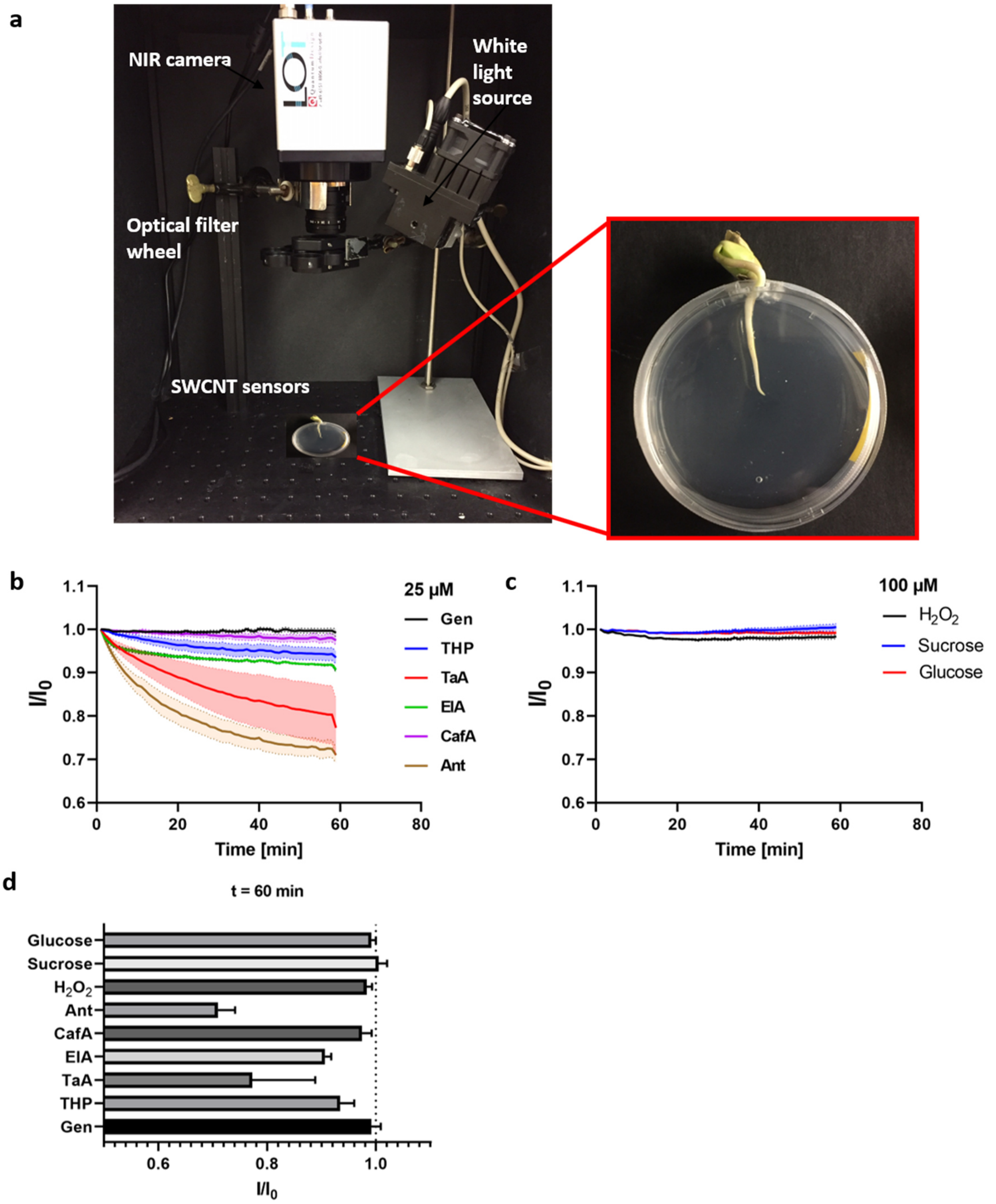

Figure S13: Imaging of sensor response during agar diffusion. a) Picture of the NIR stand-off imaging systems. A specialized InGaAs-Camera detects the fluorescence emission of the SWCNT sensors, incorporated into a agar medium (see close up image). b) PEG-PL-SWCNTs in 0.4 % culture medium agar (Murashige-Skoog-Medium) were challenged with different prominent polyphenols or possible interfering substances during NIR stand-off imaging. Nanosensor responses to 25 µM of different polyphenol compounds (line = mean, SD = pale boundaries, n = 3). c) Sensor response to potential interfering substances such as H_2O_2, mono- or disaccharides. Four times higher concentrations with 100 µM did not change the nanosensors (line = mean, SD = pale boundaries, n = 3). d) Plotted NIR-fluorescence changes of the PEG-PL-SWCNTs agar after 60 min (mean ± SD, n = 3). The sensors response to large (for rhizosphere conditions even unphysical high) H_2O_2 concentrations is within a range of ~3%, far less than the detected polyphenol secretion after elicitor stimulus. However, a small contribution of released H_2O_2 on a short timescale can not be excluded, but should not affect the overall sensing outcome (as seen in Figure 4 e,f).

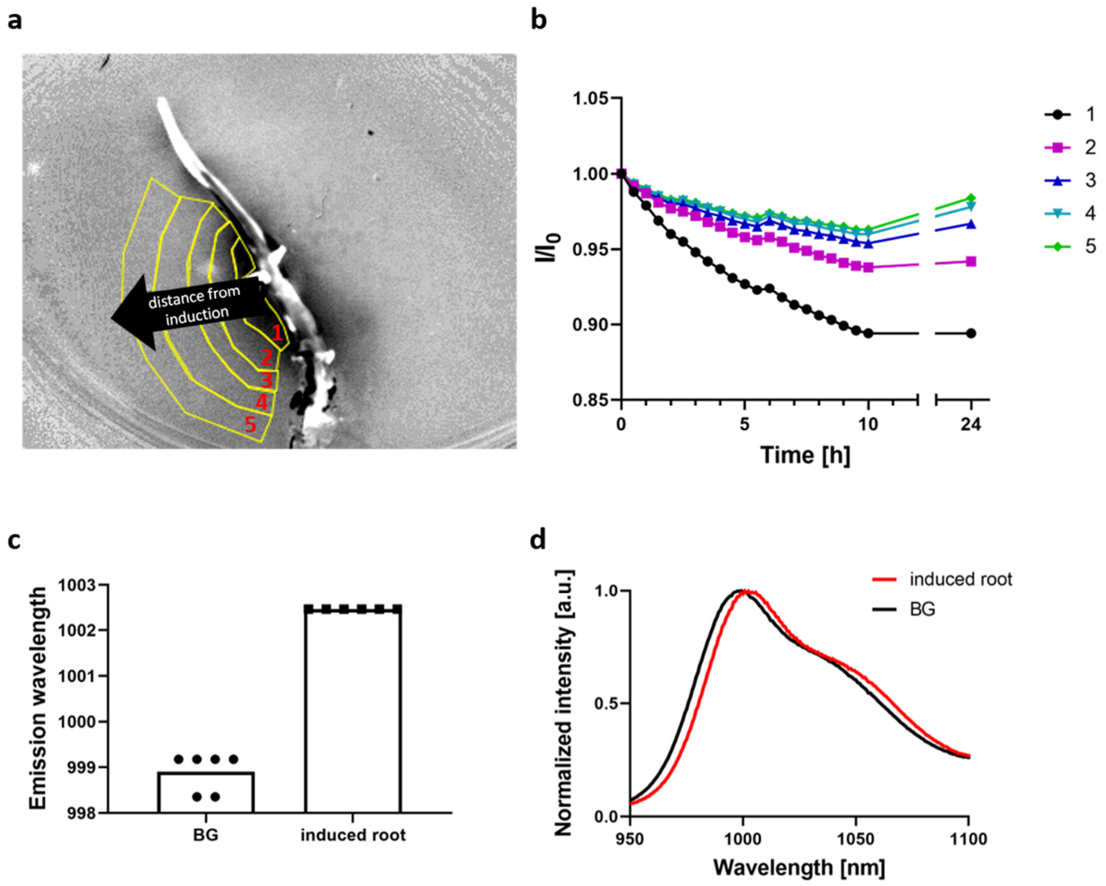

Figure S14: Elicitor induced polyphenol release from soybean seedlings. a) NIR fluorescence image of the *G. max* seedling shown in Figure 4d (10 h after induction). Different regions of interests (ROI) in the nanosensor-agar are marked, with an increasing distance from the induced root position. b) Mean intensity changes (I/I_0) of the associated ROI 1-5 are plotted over time, suggesting a constantly increasing release of polyphenols for 5-8 h after stimulus. c) Measuring the photoluminescence of the nanosensors at six random spots in the background (BG) and close to the induced region (ROI 1, labeled as 'induced root'), a clear redshift of the nanosensor emission becomes visible (mean, n = 6). d) Normalized mean fluorescence spectra of the described positions in (c) visualize the redshifted maxima. The fluorescence reduction during NIR stand-off imaging correlates therefore also with a shift in the emission spectra, as expected from polyphenol – nanosensor interaction. Moreover, with specialized optical systems also spectral-resolved imaging[19] would be possible, likely increasing sensitivity of the presented approach. However, implementation of this technique for scientific of agricultural research and crop production would be more straightforward with the simple NIR imaging system we presented. In general, Polyphenol concentrations in the soil depend on several abiotic and biotic factors. The amounts can change with the season[20], plant age[21] and nutrient supply[22]. These factors, however, are predictable and the changes are bound to occur slowly with an overall low fold change (≤ 2).[20] Induction after pathogen attack on the other hand leads to a drastic increase of defensive compounds within 4-72 h. So, soybean root cell cultures exposed to fungal pathogen (Fusarium) showed 30x increase in glyceollin concentration.[23] Overall, the sensor response from pathogen-mediated fast and drastic increase in polyphenol levels should not be biased by other factors. These findings and visualization of spatiotemporal polyphenol release are therefore in agreement with previous studies, which measured the bulk accumulation of glyceollin and THP over the course of 32 h[24] or *via* radioimmunoassay analysis.[18]

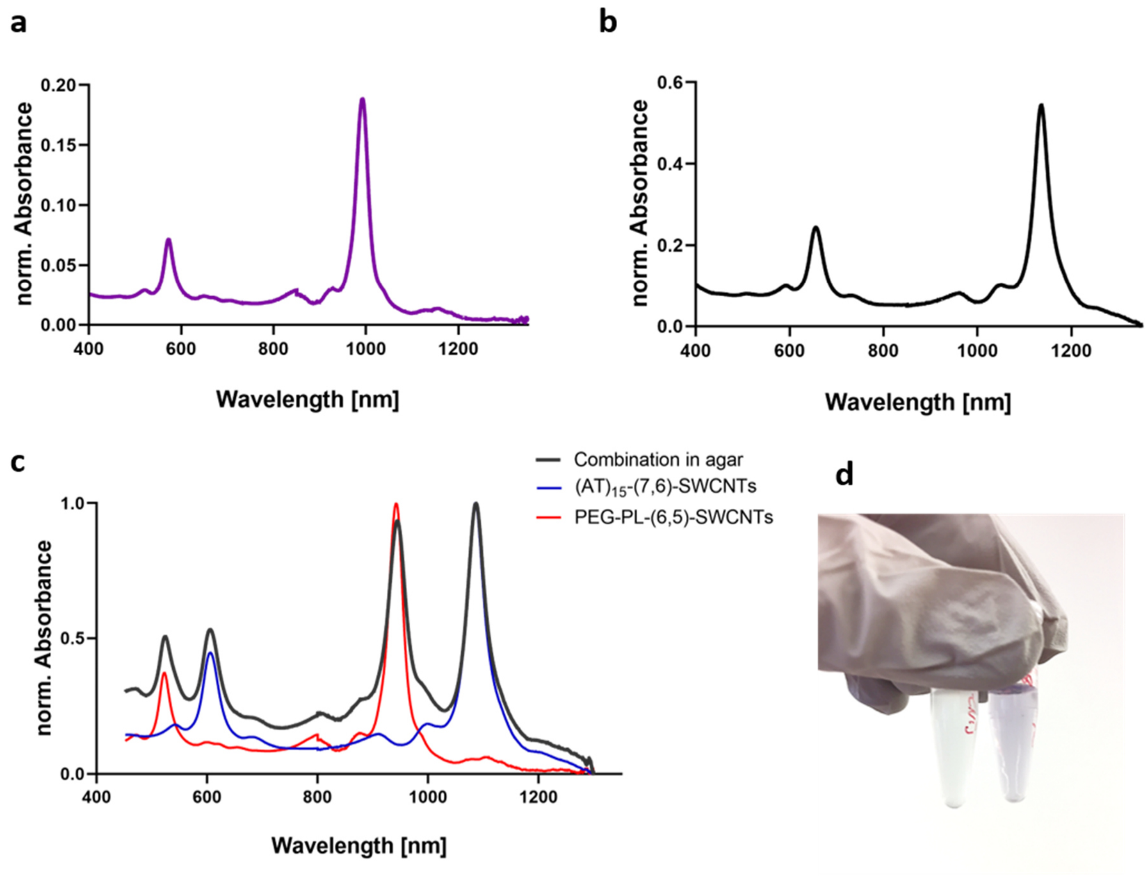

Figure S15: Separated SWCNT fractions for hyperspectral sensing. a) Absorbance spectra of PEG-PL-(6,5)-SWCNTs. b) Absorbance spectra of $(AT)_{15}$-(7,6)-SWCNTs. c) Normalized absorbance spectra of the purified and specifically modified SWCNT chiralities, measured independently and combined in agar. d) Photograph of Eppendorf tubes, containing $(AT)_{15}$-(7,6)-SWCNTs (left) and PEG-PL-(6,5)-SWCNTs (right).

References

[1] R. Nißler, F. A. Mann, P. Chaturvedi, J. Horlebein, D. Meyer, L. Vukovic, S. Kruss, *J. Phys. Chem. C* **2019**, *123*, 4837–4847.
[2] K. Welsher, Z. Liu, S. P. Sherlock, J. T. Robinson, Z. Chen, D. Daranciang, H. Dai, *Nat. Nanotechnol.* **2009**, *4*, 773–780.
[3] F. Schöppler, C. Mann, T. C. Hain, F. M. Neubauer, G. Privitera, F. Bonaccorso, D. Chu, A. C. Ferrari, T. Hertel, *J. Phys. Chem. C* **2011**, *115*, 14682–14686.
[4] H. Li, G. Gordeev, O. Garrity, S. Reich, B. S. Flavel, *ACS Nano* **2019**, *13*, 2567–2578.
[5] R. Nißler, L. Kurth, H. Li, A. Spreinat, I. Kuhlemann, B. S. Flavel, S. Kruss, *Anal. Chem.* **2021**, *93*, 6446–6455.
[6] R. Nißler, O. Bader, M. Dohmen, S. G. Walter, C. Noll, G. Selvaggio, U. Groß, S. Kruss, *Nat. Commun.* **2020**, *11*, 1–12.
[7] R. Bergomaz, M. Boppre, *J. Lepid. Soc.* **1986**, *40*, 131–137.
[8] J. Fliegmann, G. Schüler, W. Boland, J. Ebel, A. Mithöfer, *Biol. Chem.* **2003**, *384*, 437–446.
[9] C. Grajeda-Iglesias, M. C. Figueroa-Espinoza, N. Barouh, B. Baréa, A. Fernandes, V. De Freitas, E. Salas, *J. Nat. Prod.* **2016**, *79*, 1709–1718.
[10] A. Mithöfer, A. A. Bhagwat, M. Feger, J. Ebel, *Planta* **1996**, *199*, 270–275.
[11] A. Mithöfer, A. A. Bhagwat, D. L. Keister, J. Ebel, *Zeitschrift fur Naturforsch. - Sect. C J. Biosci.* **2001**, *56*, 581–584.
[12] N. D. Lackus, A. Müller, T. D. U. Kröber, M. Reichelt, A. Schmidt, Y. Nakamura, C. Paetz, K. Luck, R. L. Lindroth, C. P. Constabel, S. B. Unsicker, J. Gershenzon, T. G. Köllnera, *Plant Physiol.* **2020**, *183*, 137–151.
[13] E. A. Ainsworth, K. M. Gillespie, *Nat. Protoc.* **2007**, *2*, 875–877.
[14] D. P. Salem, X. Gong, A. T. Liu, V. B. Koman, J. Dong, M. S. Strano, *J. Am. Chem. Soc.* **2017**, *139*, 16791–16802.
[15] J. Moilanen, J. Sinkkonen, J. P. Salminen, *Chemoecology* **2013**, *23*, 165–179.
[16] D. M. O. Serna, J. H. I. Martínez, *Molecules* **2015**, *20*, 17818–17847.
[17] D. Fracassetti, C. Costa, L. Moulay, F. A. Tomás-Barberán, *Food Chem.* **2013**, *139*, 578–588.
[18] M. G. Hahn, A. Bonhoff, H. Grisebach, *Plant Physiol.* **1985**, *77*, 591–601.
[19] D. Roxbury, P. V. Jena, R. M. Williams, B. Enyedi, P. Niethammer, S. Marcet, M. Verhaegen, S. Blais-Ouellette, D. A. Heller, *Sci. Rep.* **2015**, *5*, 1–6.
[20] T. Sosa, C. Valares, J. C. Alías, N. C. Lobón, *Plant Soil* **2010**, *337*, 51–63.
[21] S. Cesco, T. Mimmo, G. Tonon, N. Tomasi, R. Pinton, R. Terzano, G. Neumann, L. Weisskopf, G. Renella, L. Landi, P. Nannipieri, *Biol. Fertil. Soils* **2012**, *48*, 123–149.
[22] S. Cesco, G. Neumann, N. Tomasi, R. Pinton, L. Weisskopf, *Plant Soil* **2010**, *329*, 1–25.
[23] V. V. Lozovaya, A. V. Lygin, O. V. Zernova, S. Li, G. L. Hartman, J. M. Widholm, *Plant Physiol. Biochem.* **2004**, *42*, 671–679.
[24] P. Moesta, H. Grisebach, *Nature* **1980**, *286*, 710–711.

Author Contributions

R.N. and S.K. designed and conceived the research with input from J.P.G. S.K. coordinated the project. R.N., F.D. and L.K performed chemical sensing experiments and separated (6,5)-SWCNTs. A.T.M., E.G.C. and A.M. performed plant cultivation, extraction and related analysis experiments. H.L. and B.S.F. performed (7,6)-SWCNT separation. All authors contributed to the writing of the manuscript and analysis of data. All authors have given approval to the final version of the manuscript.
